# The Impact of Extended CO_2_ Cross Sections on Temperate Anoxic Planet Atmospheres

**DOI:** 10.3847/1538-4357/adaaf0

**Published:** 2025-02-14

**Authors:** Wynter Broussard, Edward W. Schwieterman, Clara Sousa-Silva, Grace Sanger-Johnson, Sukrit Ranjan, Olivia Venot

**Affiliations:** 1 Department of Earth and Planetary Sciences, University of California, Riverside, CA 92521, USA; abrou009@ucr.edu; 2 Blue Marble Space Institute of Science, Seattle, WA 98104, USA; 3 Bard College, 30 Campus Rd, Annandale-On-Hudson, NY 12504, USA; 4 Institute of Astrophysics and Space Sciences, Rua das Estrelas, 4150-762 Porto, Portugal; 5 Department of Physics and Astronomy, Michigan State University, East Lansing, MI 48824, USA; 6 University of Arizona, Lunar and Planetary Laboratory/Department of Planetary Sciences, Tucson, AZ 85721, USA; 7 Université Paris Cité and Univ. Paris Est Creteil, CNRS, LISA, F-75013 Paris, France

## Abstract

Our interpretation of terrestrial exoplanet atmospheric spectra will always be limited by the accuracy of the data we use as input in our forward and retrieval models. Ultraviolet molecular absorption cross sections are one category of these essential model inputs; however, they are often poorly characterized at the longest wavelengths relevant to photodissociation. Photolysis reactions dominate the chemical kinetics of temperate terrestrial planet atmospheres. One molecule of particular importance is CO_2_, which is likely present in all terrestrial planet atmospheres. The photolysis of CO_2_ can introduce CO and O, as well as shield tropospheric water vapor from undergoing photolysis. This is important because H_2_O photolysis produces OH, which serves as a major reactive sink to many atmospheric trace gases. Here, we construct CO_2_ cross-section prescriptions at 195 K and 300 K extrapolated beyond 200 nm from measured cross sections. We compare results from the implementation of these new cross sections to the most commonly used CO_2_ prescriptions for temperate terrestrial planets with Archean-like atmospheres. We generally find that the observational consequences of CO_2_ dissociation beyond 200 nm are minimal so long as our least conservative (highest opacity) prescription can be ruled out. Moreover, implementing our recommended extended CO_2_ cross sections does not substantially alter previous results that show the consequential photochemical impact of extended H_2_O cross sections.

## 1. Introduction

The search for life outside our solar system is centered around planets like Earth: small, rocky planets with secondary atmospheres (L. Kaltenegger 2017; E. W. Schwieterman et al. 2018). In the current state of exoplanet science, JWST represents a prime opportunity to observe and characterize the atmospheres of these Earth-sized terrestrial exoplanets that orbit in the habitable zones of their host stars (TRAPPIST-1 JWST Community Initiative et al. 2024; E. M. R. Kempton & H. A. Knutson 2024). However the most compelling targets for observations by JWST are those planets orbiting M-type stars (C. V. Morley et al. 2017; E. M. May et al. 2023). In 2021, the Astronomy & Astrophysics Decadal Survey, which highlights the scientific priorities, opportunities, and funding recommendations for the next decade, listed identifying and characterizing terrestrial exoplanets as a key goal (National Academies of Sciences, Engineering, and Medicine 2021). With this, one of the survey's top priorities is the development of the Habitable Worlds Observatory (HWO). HWO will be optimized for observing reflected light from small planets orbiting Sun-like host stars in the IR, optical, and UV (E. Mamajek & K. Stapelfeldt 2024).

We can use photochemical modeling to predict the possible atmospheres of some of the many exoplanets that have been discovered to date (J. Krissansen-Totton et al. 2018; A. P. Lincowski et al. 2018; R. Hu et al. 2021; N. Madhusudhan et al. 2023; V. S. Meadows et al. 2023). Forward modeling is also critical in helping to inform the development and design specifications of future exoplanet observing missions, as well as in interpreting observed exoplanet spectra (T. P. Greene et al. 2016; M. H. Currie et al. 2023; N. F. Wogan et al. 2024). The models used in photochemical studies require accurate inputs, including chemical reaction rates, molecular absorption cross sections, stellar spectra, dry and wet deposition rates, mixing parameterizations, and more. Models that are set with specific conditions in mind (often for the Earth or other solar system worlds) can err in their predictions when they are used to model atmospheres with substantially different boundary conditions. Additionally, models that combine incompatible photochemical inputs can yield erroneous results, and can lead to conflicting interpretations of observations.

Previous studies have shown that updates to the H_2_O mid-UV (MUV; 200–300 nm) absorption cross sections can meaningfully impact predictions of trace gas chemistry on anoxic, temperate, terrestrial exoplanets (S. Ranjan et al. 2020; W. Broussard et al. 2024). Past H_2_O cross-section prescriptions cut off at ∼200–208 nm, where H_2_O’s opacity falls below the typical scattering opacity of the atmosphere. However, in thick anoxic atmospheres, the 200–240 nm range is critical for the atmospheric chemistry (J.-S. Wen et al. 1989). This is because stellar MUV photons penetrate further into the H_2_O-rich troposphere, whereas the higher-energy far-UV (<200 nm) photons are stopped from reaching the troposphere by overlying CO_2_. With newly measured H_2_O cross sections that extend into the MUV, more H_2_O photolysis following Equation (1) will occur, generating more of the highly reactive OH radical.

\begin{eqnarray*}{{\mathrm{H}}}_{2}{\mathrm{O}}+h\nu \to {\mathrm{H}}+{\mathrm{OH}}\end{eqnarray*}

OH is an effective sink for many atmospheric trace gases, such as CO and CH_4_. With more OH available to remove these gases, the extended H_2_O cross sections lead to lower predicted volume mixing ratios of atmospheric trace gases for these species.

There are two primary channels for the photolysis of CO_2_:

\begin{eqnarray*}{{\mathrm{CO}}}_{2}+h\nu \to {\mathrm{CO}}{(}^{1}{{\mathrm{\Sigma }}}^{+})+{\mathrm{O}}{(}^{1}{\mathrm{D}})\end{eqnarray*}

\begin{eqnarray*}{{\mathrm{CO}}}_{2}+h\nu \to {\mathrm{CO}}{(}^{1}{{\mathrm{\Sigma }}}^{+})+{\mathrm{O}}{(}^{3}{\mathrm{P}}).\end{eqnarray*}

**Figure 1.**
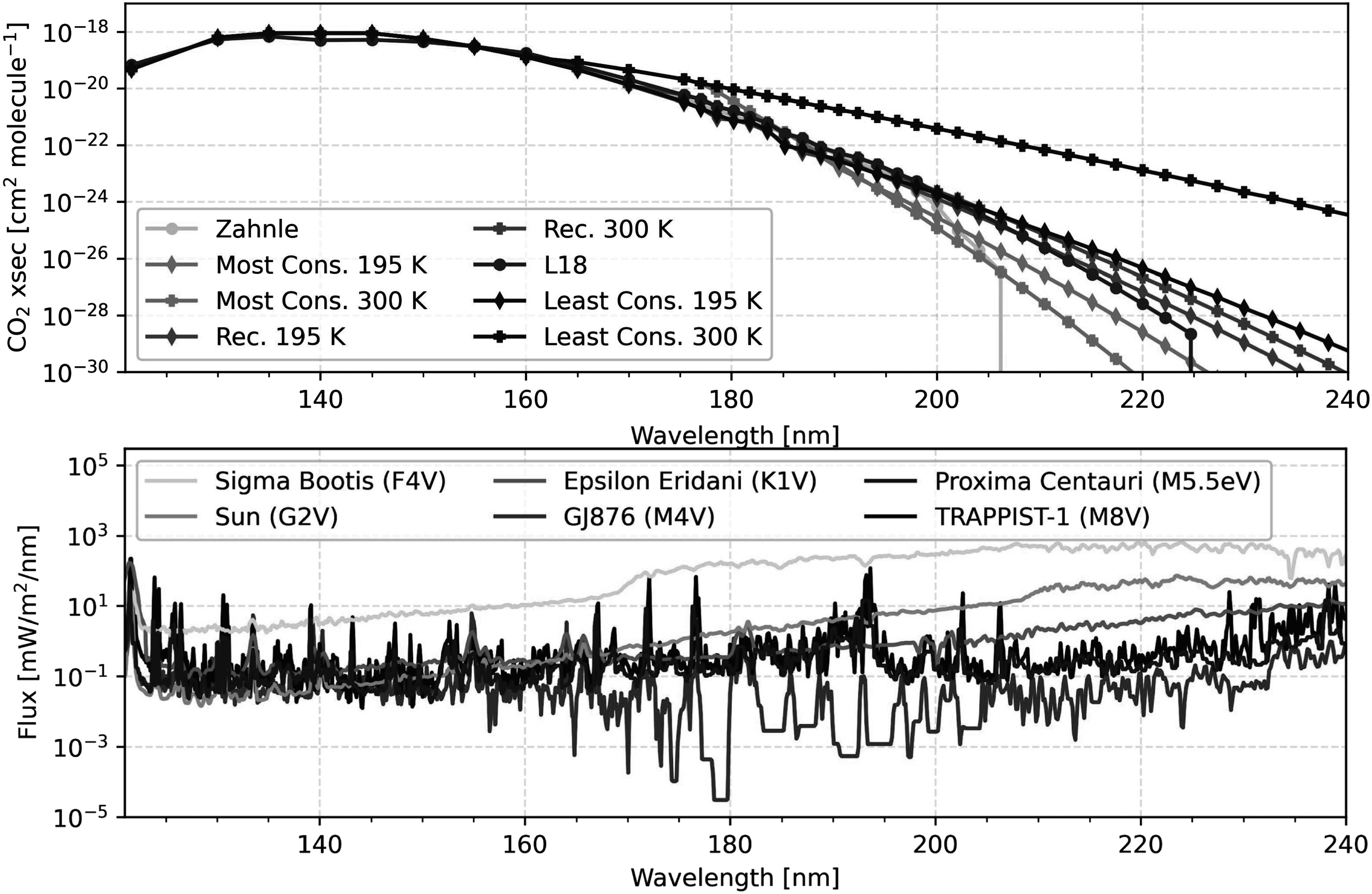
Top: CO_2_ cross-section prescriptions from 120 to 240 nm. Bottom: spectral energy distribution of the stars modeled in this study, from 120 to 240 nm.

The channel in Equation (2) has a quantum limit at 167.2 nm while the channel in Equation (3) has a quantum limit of 227.5 nm, representing the energy needed to break the CO–O bond in the ground state (J. A. Schmidt et al. 2013). Beyond this quantum limit, only forbidden transitions can occur, but the accumulation of these forbidden transitions can still potentially add to the opacity of the cross sections. For this reason, as seen in Figure 1, our extrapolation prescriptions continue beyond 227.5 nm, to account for this cumulative effect. Termination wavelengths for CO_2_ cross sections vary from model to model. Most databases currently recommend a cutoff near ∼200 nm (S. P. Sander et al. 2011), which was used by both S. Ranjan et al. (2020) and W. Broussard et al. (2024) and lies predictably near the wavelength where scattering opacities begin to overwhelm dissociation opacities (D. Ityaksov et al. 2008). A. P. Lincowski et al. (2018) used a log-extrapolated cutoff at 225 nm when modeling the photochemistry of the TRAPPIST-1 planets. The public version of the Atmos photochemical model (G. Arney et al. 2016) uses a cutoff prescription of ∼208 nm, between these end-members.

CO_2_ inputs are particularly consequential, as our definition of a traditionally habitable, Earth-like world is predicated on an N_2_–CO_2_–H_2_O atmosphere with a negative carbon feedback cycle (R. K. Kopparapu et al. 2013). Such worlds are also expected as a consequence of planetary outgassing (F. Gaillard & B. Scaillet 2014). The results of W. Broussard et al. (2024) showing the impact of the extended H_2_O cross sections into the MUV were based on simulations that did not include CO_2_ absorption in this wavelength region. If CO_2_ has appreciable absorption in the MUV, MUV photons will be stopped from reaching the H_2_O-rich troposphere by the overlying CO_2_, thus the impact of the extended H_2_O cross sections could be minimized. Likewise the introduction of CO, O(^3^P), and O(^1^D) from the photolysis of CO_2_ can consume OH radicals produced via H_2_O photolysis, further contributing to the suppression of OH and minimizing the impact of the H_2_O cross sections. Furthermore, the photochemical production of CO, O(^3^P), and O(^1^D) has a variety of other spectral and chemical implications, including those assessing the potential for abiotic O_2_ and O_3_ accumulation and resulting spectral signatures that could constitute a false positive for life (e.g., P. Gao et al. 2015; C. Harman et al. 2015; E. W. Schwieterman et al. 2016; S. Ranjan et al. 2020). It is therefore essential to understand the degree to which corresponding extended CO_2_ cross sections would impact those earlier results. In the absence of laboratory measurements of CO_2_’s cross sections at room temperature in the MUV, we can use extrapolations to predict these data. In this paper, we use three cross-section extrapolations (described further in Section 2.1) in addition to the prescription used by A. P. Lincowski et al. (2018; the “L18” prescription), and the prescription used in the public version of Atmos (the “Zahnle” prescription; G. Arney et al. 2016). The three extrapolations, shown in Figure 1, include a most conservative extrapolation (which has the least opacity and thus leads to the least CO_2_ photolysis), a least conservative extrapolation (which has the most opacity, leading to the most CO_2_ photolysis), and a recommended extrapolation (which lies between these end-members).

In this paper, we test the impact of these prescriptions for CO_2_’s cross sections in temperate, anoxic, terrestrial planet atmospheres, and make recommendations for harmonizing the extended CO_2_ cross-section inputs in community models. In Section 2, we describe the construction of the extended CO_2_ cross sections via empirical and theoretical sources (a “best-fit” range), comparing them quantitatively to current prescriptions. Additionally we describe the photochemical and spectral models, as well as the planetary scenario used to test the sensitivity of these inputs under a range of CH_4_ surface fluxes and CO_2_ surface volume mixing ratios, for FGKM-type host stars. In Section 3 we report our results, including impacts on trace gas species and spectral observables. We also revisit the H_2_O cross-section sensitivity tests of W. Broussard et al. (2024) with these updated CO_2_ cross sections. We discuss the implications of our results in Section 4 and conclude in Section 5.

## 2. Methods

### 2.1. Cross-section Prescriptions

The CO_2_ cross sections were prepared using a similar prescription to the extrapolation of the H_2_O cross sections presented by S. Ranjan et al. (2020) and used by W. Broussard et al. (2024). Under a first-order assumption of a linear-log loss of opacity toward dissociation, over large wavenumber bins, several possible extrapolations were proposed based on existing experimental data measured primarily at wavelengths ≤200 nm (Table 1). Our extrapolations were created by following the gradient, calculated from

\begin{eqnarray*}({\mathrm{log}}({\sigma }_{{\lambda }_{b}})-{\mathrm{log}}({\sigma }_{{\lambda }_{a}}))/{\mathrm{log}}({\sigma }_{{\lambda }_{b}})\end{eqnarray*}

from the baseline of the clear rovibrational structures present in the measured data, where *σ* is the absorption cross section. We selected structures that were toward the end of the measured data, and therefore toward the end of the instrument sensitivity, but otherwise at a long wavelength so as to create a more representative logarithmic trend. The range of opacities in the predicted cross sections come primarily from the variation in accuracy and precision of the measured data used as a basis for the theoretical extrapolations. A secondary reason for the variation in extrapolations derives from the range of possible linear-log gradients that can be predicted from the overall opacity-loss trend in the rovibrational structure of the measured data. For every presented temperature, three cross-section extrapolations were created (recommended, and most/least conservative) to allow for a scientifically meaningful sensitivity analysis of the impact of these cross sections on the photochemical models explored in this work. Note that “least conservative” extrapolations have the highest dissociation opacity at wavelengths ≥200 nm, while the “most conservative” extrapolations have the lowest dissociation opacity at wavelengths ≥200 nm. Notably, due to the higher complexity of rovibrational features measured at low temperatures, the extrapolated linear-log gradients did not always decrease with lower temperatures, despite there being no physical reason to expect higher opacities at lower temperatures. For example, the most conservative extrapolation at 300 K is well below all of the extrapolations at 195 K as seen in Figure 1. However, not all of the extrapolations show this artifact, as other higher-temperature extrapolations exhibit higher opacities as expected. Nonetheless, this exception only applies to the extreme (most and least conservative) extrapolations, not to the recommended opacities.

**Table 1:** CO_2_ Cross-section Experimental Data Used for Extrapolations

Temperature	Wavelength Range	Extrapolation	Data Source
(K)	(nm)		
195	163–192.5	recommended	O. Venot et al. (2018)
195	163–192.5	most conservative	W. Parkinson et al. (2003)
195	163–192.5	least conservative	W. Parkinson et al. (2003)
295	163–200	recommended	W. Parkinson et al. (2003)
300	115.3–187.5	most conservative	O. Venot et al. (2018)
300	115–200	least conservative	O. Venot et al. (2018)

It is worth noting that the empirically calibrated but theoretical CO_2_ cross sections used in this work should be considered only as a reasonable substitution given the absence of data at those wavelengths—valuable for sensitivity analyses, but not an adequate replacement for accurate measured data, which we recommend should be funded and obtained. It is reasonable to assume, however, that the true cross sections, once measured, will fall in the range of theoretical extrapolations measured here.

Table 1 lists the sources used to create the theoretical extrapolations of the CO_2_ cross sections. The cross-section extrapolations are available at https://github.com/abrou009/broussard_2025_co2.

### 2.2. Photochemical Model Description

This research employs the photochemical model Atmos (G. Arney et al. 2016, 2018; R. C. Felton et al. 2022; E. W. Schwieterman et al. 2022), the same one-dimensional model as was used by W. Broussard et al. (2024). For a more detailed description of the photochemical portion of Atmos, see Section 2.2 of W. Broussard et al. (2024). Also contained within Atmos is the radiative–convective climate model, Clima. Clima was first developed to model high CO_2_ concentrations in the early Earth's atmosphere (J. F. Kasting & T. P. Ackerman 1986), but has since received updates to model a broader range of climate scenarios (G. Arney et al. 2016). Clima can be run independently of the photochemistry portion of Atmos, or both portions can be run in the coupled mode. To use Atmos in the photochemistry–climate coupling mode, first the photochemical portion is run to convergence. The altitude-dependent volume mixing ratios of relevant gas species are returned once convergence is reached, and this output is used as the initial input into the climate portion of the model. Clima updates the water vapor and temperature profiles, which are then returned to the photochemistry portion of Atmos, and this cycle repeats until both the photochemistry and climate models have converged.

In this research, our utilization of Atmos differs from that of W. Broussard et al. (2024) in two ways: in the chosen temperature–pressure profile and in the implementation of temperature-dependent cross sections for CO_2_. The temperature–pressure profile used in this research has a surface temperature of 288 K (Earth's average modern surface temperature) and was calculated to be climatically self-consistent up to 68.25 km, which represents the model top of the climate simulations. Above this height the atmosphere takes on an isothermal profile with a temperature of 182 K. This profile represents a reasonable approximation of a temperature–pressure profile for the Archean Earth, and is used for each of the stars modeled in this study to isolate the effects of the changing CO_2_ cross sections and one other variable (e.g., CH_4_ production rate, CO_2_ mixing ratio, stellar spectrum, etc.). For all planets except those orbiting Proxima Centauri and TRAPPIST-1, the stellar spectra were scaled so that the planet received a top-of-atmosphere flux equal to the solar constant. We note that past climate modeling has shown that planets that receive an Earth-average insolation flux will have a different surface temperature depending on the spectral energy distribution of the host star (A. A. Segura et al. 2005; G. Arney et al. 2018; A. D. Del Genio et al. 2019). M dwarf host stars produce more red and infrared light than stars of earlier types, which is more easily transmitted (not scattered) through a planetary atmosphere. Consequently, planets orbiting M-type host stars will have higher surface temperatures for a given bolometric flux than planets orbiting G- or F-type host stars, where proportionally more of the radiation received at the top of the atmosphere is scattered away, increasing planetary albedo. As the changing tropospheric water content resulting from the changing surface temperature would have a strong impact on our results, we use the same surface temperature for all planets regardless of the stellar type of the host star as a simplifying assumption, allowing us to facilitate direct intercomparisons between the host stars and CO_2_ cross sections.

We can factor in some of the temperature-dependent nature of the photochemical cross sections, and account for the majority of the altitude-dependent temperature variation anticipated for a habitable planet's atmosphere, by including an interpolation between the prescriptions at each provided temperature. Thus, we use a temperature-dependent linear interpolation between the 195 K and 300 K cross-section data sets. For temperatures greater than 300 K, the 300 K cross sections would be assumed, and the 195 K cross sections would be assumed for temperatures less than 195 K.

### 2.3. Spectral Model Description

This research employs the same spectral model as W. Broussard et al. (2024), the Spectral Mapping Atmospheric Radiative Transfer code (SMART; V. S. Meadows & D. Crisp 1996; D. Crisp 1997). For a more detailed description of SMART, see Section 2.3 of W. Broussard et al. (2024). For the purpose of this research, we have modeled spectral scenarios assuming Sigma Boötis, the Sun, and TRAPPIST-1 as the host star. We give the stellar and planetary parameters assumed in Table 2. Planetary parameters were chosen for Sigma Boötis, Epislon Eridani, and GJ 876 as a host star so that the planet would have a solar constant equal to the Earth's current solar constant. For Proxima Centauri and TRAPPIST-1 as the host stars, the planetary parameters of Proxima Centauri b and TRAPPIST-1 e were assumed, respectively.

**Table 2:** Stellar and Planetary Properties

Star	Spectral Type	*T*_eff_	Luminosity	Stellar Radius	Distance	Planetary Radius	Planet–Star Distance
		(K)	(*L*_⊙_)	(*R*_⊙_)	(pc)	(*R*_⊕_)	(au)
*σ* Boötis	F4V	6435	3.1541	1.4307	15.8	1	1.776
Sun	G2V	5780	1	1	⋯	1	1
*ϵ* Eridani	K1V	5039	0.32	0.735	3.2	1	0.562
GJ 876	M4V	3129	0.0122	0.3761	4.69	1	0.110
Proxima Centauri	M5.5eV	2992	0.001567	0.147	1.3	1	0.049
TRAPPIST-1	M8V	2559	0.000524	0.117	12.1	0.91	0.029

### 2.4. Stellar Spectra

To show how our results vary with the type of host star, we chose to conduct these sensitivity tests with six different main-sequence host stars, with stellar parameters listed in Table 2. These stars are: the F-type star *σ* Boötis (A. Segura et al. 2003), the Sun, which is a G-type star (G. Thuillier et al. 2004), the K-type star *ϵ* Eridani (A. Segura et al. 2003), and three M-type stars: GJ 876 (M4V) (K. France et al. 2016; A. Youngblood et al. 2016; R. O. P. Loyd et al. 2016 (v22)), Proxima Centauri (M5.5eV) (E. L. Shkolnik & T. S. Barman 2014; R. O. P. Loyd et al. 2018; S. Peacock et al. 2020), and TRAPPIST-1 (M8V) (S. Peacock et al. 2019a, 2019b). Figure 1 shows the spectral energy distribution of each of these host stars in the UV.

### 2.5. Planetary Scenario

**Figure 2.**
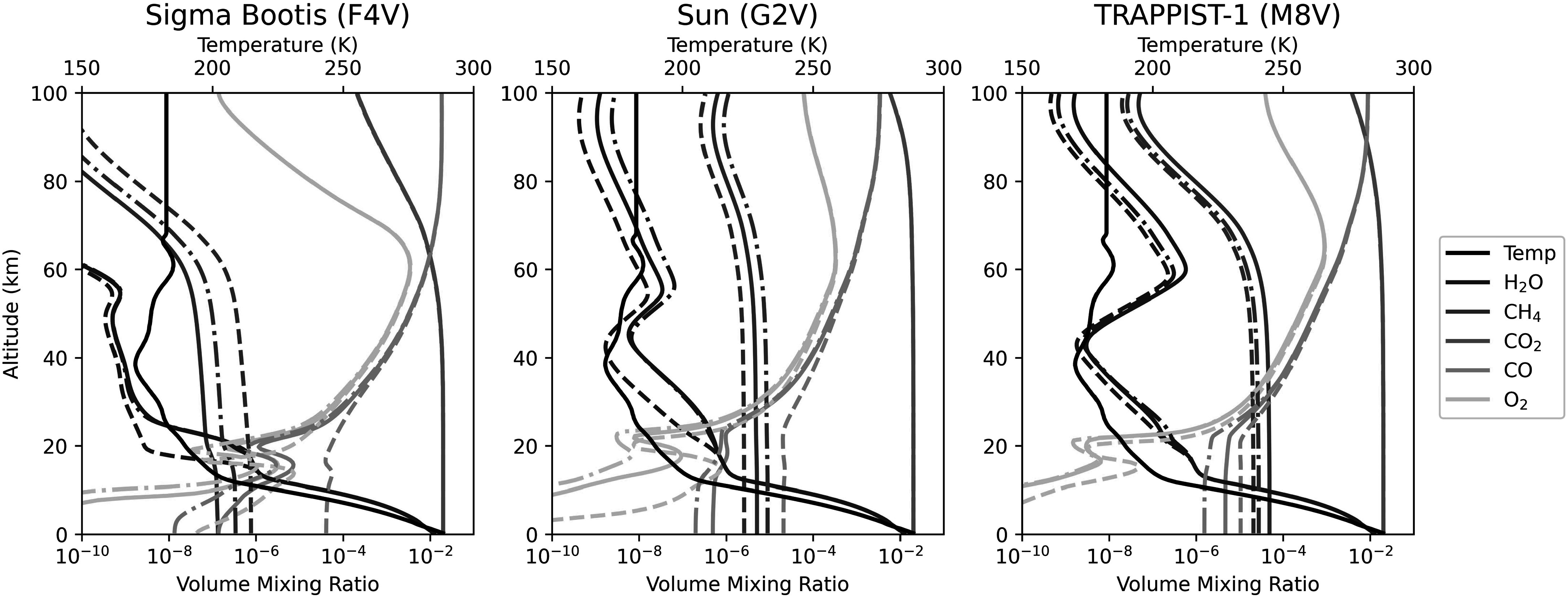
Example profile plots for planets orbiting Sigma Boötis, the Sun, and TRAPPIST-1, for a CH_4_ flux of 2 × 10^10^ molecules cm^−2^ s^−1^ and a CO_2_ volume mixing ratio of 3%. Solid lines show the altitude-dependent volume mixing ratios of key atmospheric gases modeled using the recommended CO_2_ cross sections; dashed lines are modeled using the least conservative CO_2_ cross sections, and dashed–dotted lines are modeled using the most conservative CO_2_ cross sections.

The atmospheres modeled in this work are N_2_–H_2_O–CO_2_ atmospheres. Full atmospheric boundary conditions, including surface fluxes, surface volume mixing ratios, and dry deposition velocities, can be found in Table 3 of Appendix A. Figure 2 shows three example profile plots for (from left to right) Sigma Boötis, the Sun, and TRAPPIST-1 as the host star, for a surface CH_4_ flux of 2 × 10^10^ molecules cm^−2^ s^−1^ and a CO_2_ volume mixing ratio of 3%.

For conducting the CH_4_ flux sensitivity tests, we adopt a CO_2_ mixing ratio of 3% and vary the CH_4_ surface flux from 10^8^ molecules cm^−2^ s^−1^ (∼2.67 × 10^−2^ Tmol yr^−1^), which is around the expected CH_4_ flux values for abiotic systems, such as from volcanic outgassing or serpentinization (M. A. Thompson et al. 2022), to 10^11^ molecules cm^−2^ s^−1^ (∼26.7 Tmol yr^−1^), representing a roughly Earth-like flux; Earth's current CH_4_ production levels are around 30 Tmol yr^−1^ (M. A. Thompson et al. 2022).

For the CO_2_ mixing ratio sensitivity tests, we adopt a CH_4_ flux of 10^9^ molecules cm^−2^ s^−1^ and vary the CO_2_ surface volume mixing ratio from 10^−6^, or 1 ppm, to ∼5 × 10^−1^, or about 50% CO_2_.

## 3. Results

### 3.1. Relationships between CO_2_ Cross Sections and Trace Gases

To test the sensitivity of atmospheric trace gases to the choice of CO_2_ cross-section prescription, we have conducted two sets of sensitivity tests. The first set, described in Section 3.1.1, test the response of atmospheric trace gases to CH_4_ surface flux, going from a CH_4_ flux of 10^8^ molecules cm^−2^ s^−1^ to 10^11^ molecules cm^−2^ s^−1^ (or from ∼2.67 × 10^−2^ Tmol yr^−1^ to ∼26.7 Tmol yr^−1^). As in W. Broussard et al. (2024), this parameter space was chosen to represent a gradient that goes from an abiotic value less than the upper limit of CH_4_ from serpentinization to a biotic level of CH_4_, similar to Earth's current biogenic CH_4_ flux (M. A. Thompson et al. 2022). The second set of sensitivity tests, described in Section 3.1.2, test the response of atmospheric trace gases as a function of the surface CO_2_ volume mixing ratio, varying from 10^−6^ to around 5 × 10^−1^ (or from 1 ppm to around 50% CO_2_).

#### 3.1.1. CH_4_ Flux Sensitivity Tests

**Figure 3.**
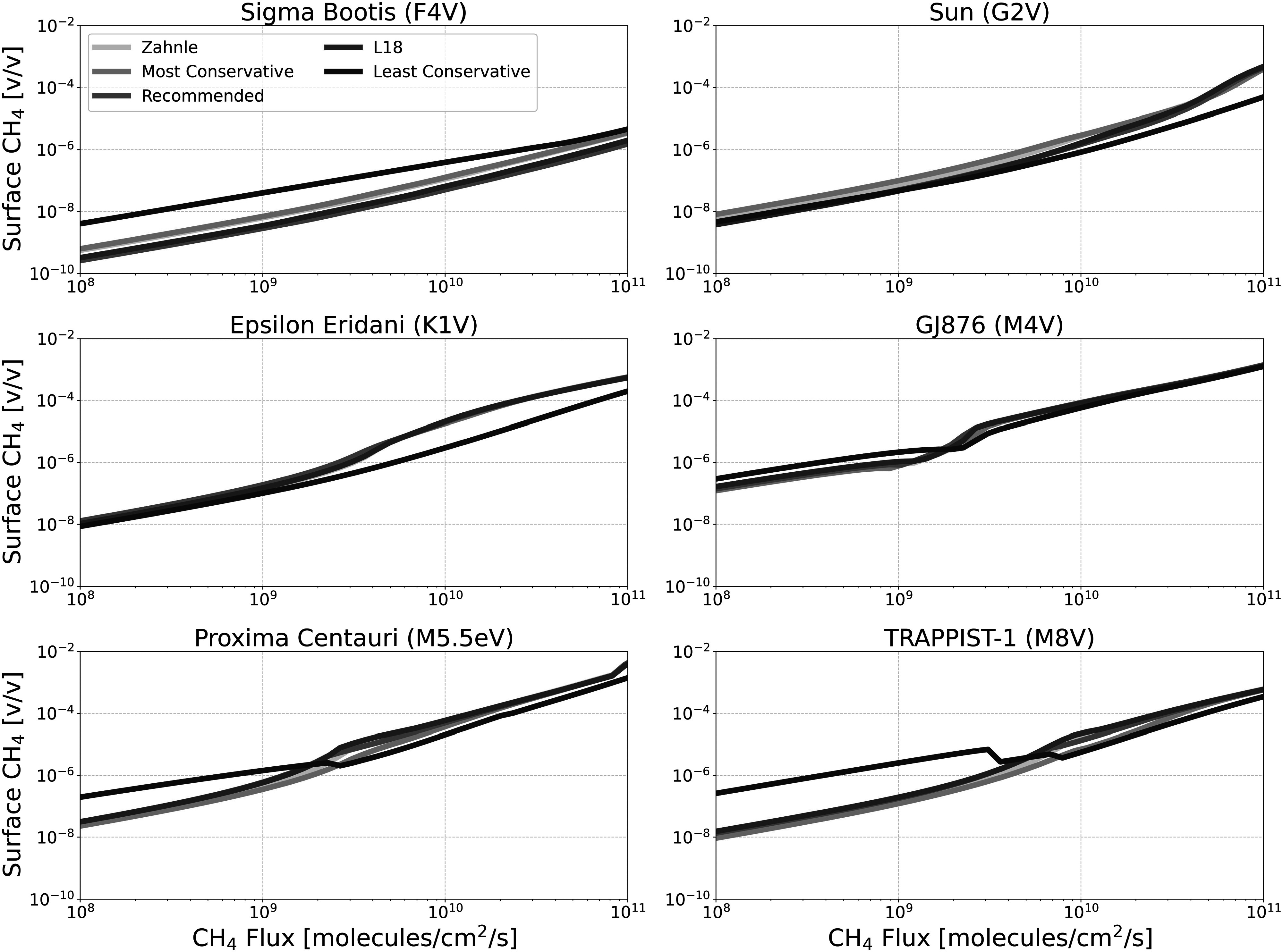
CO_2_ cross-section sensitivity test: surface CH_4_ vs. CH_4_ flux for anoxic habitable planets orbiting FGKM-type host stars.

**Figure 4.**
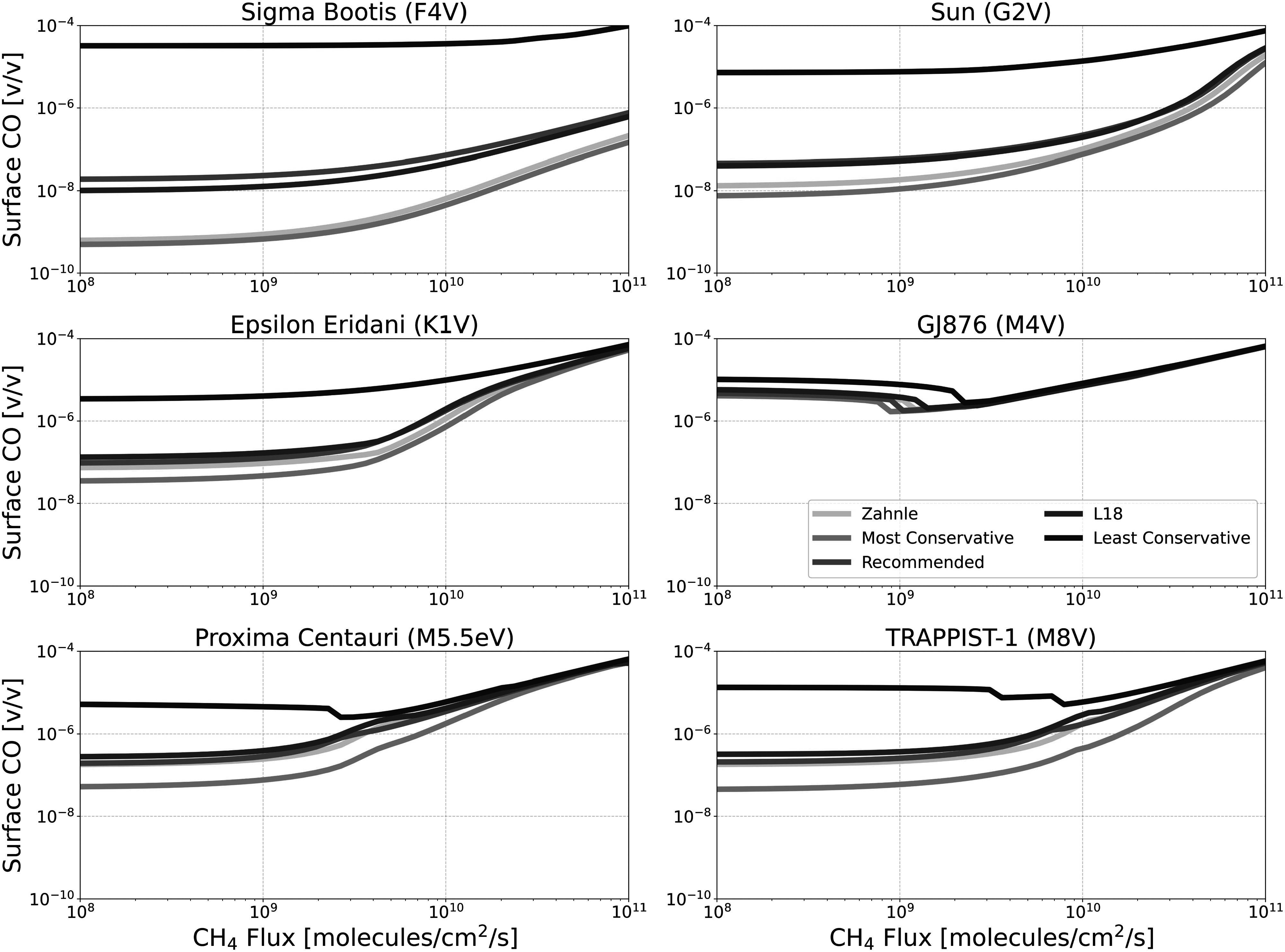
CO_2_ cross-section sensitivity test: surface CO vs. CH_4_ flux for anoxic habitable planets orbiting FGKM-type host stars.

**Figure 5.**
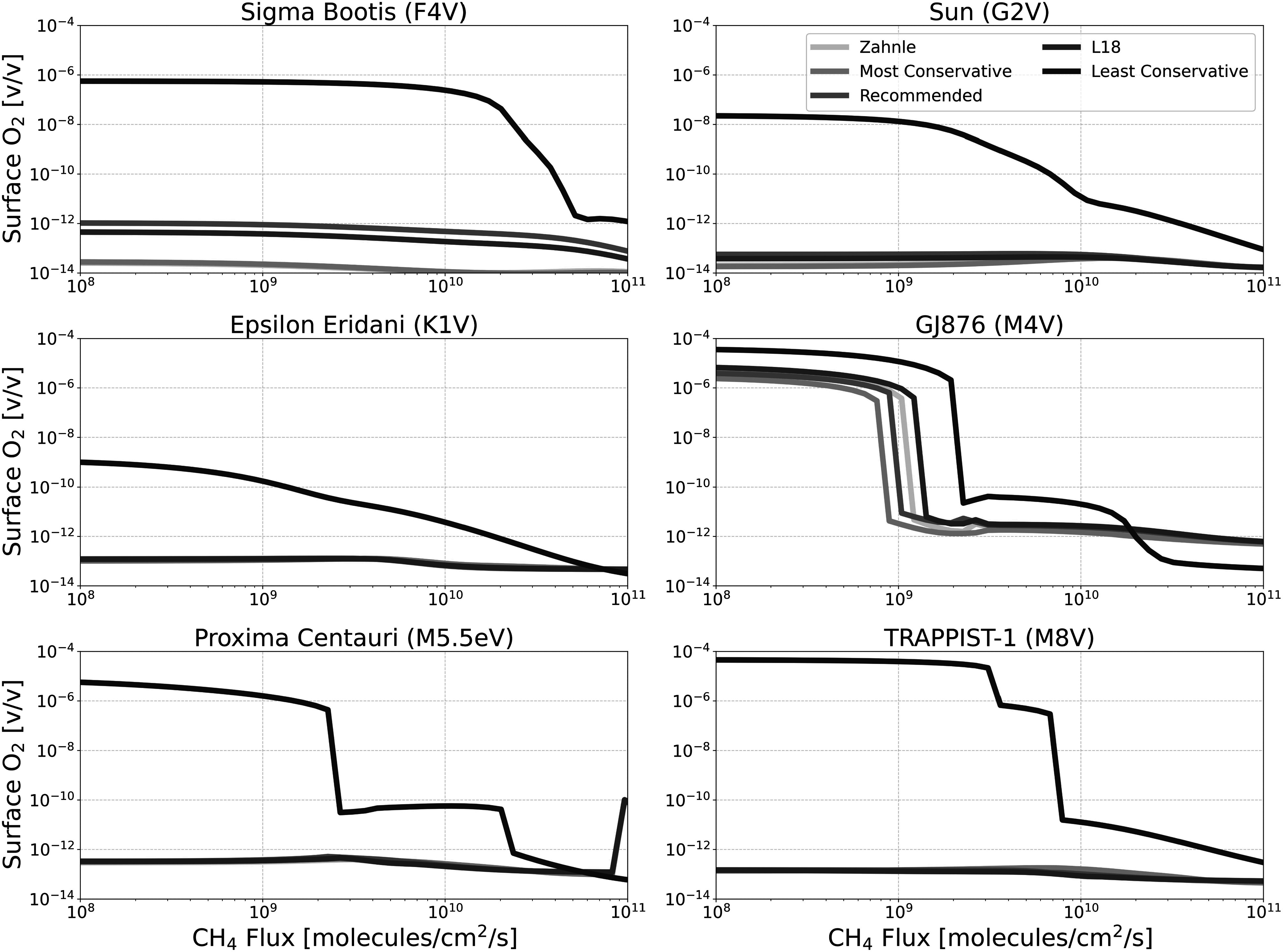
CO_2_ cross-section sensitivity test: surface O_2_ vs. CH_4_ flux for anoxic habitable planets orbiting FGKM-type host stars.

Figures 3, 4, and 5 show the impact of the various CO_2_ cross-section prescriptions on the surface volume mixing ratios of CH_4_, CO, and O_2_ as a function of CH_4_ surface flux, respectively.

In Figure 3, we see that the choice of CO_2_ cross-section prescription does not have a large impact on the resulting surface CH_4_ abundance. Four of the five cross-section prescriptions result in almost identical model predictions across the six stellar types, with one exception in the results from the least conservative prescription (which has the largest CO_2_ opacity at wavelengths >200 nm). For the F-type host star and the M5.5eV host star, at low CH_4_ fluxes, the resulting surface CH_4_ is around an order of magnitude larger than surface CH_4_ modeled using the other prescriptions; for the M8V host star, the difference is a little over an order of magnitude. Additionally, for the G-type host star at the largest CH_4_ fluxes, the surface CH_4_ modeled using the least conservative prescription diverges from models run using the other prescriptions. Although there is less OH produced from H_2_O photolysis for models run using the least conservative prescription, there is also more O(^3^P) and O(^1^D) generated from the increased CO_2_ photolysis. Thus, this increased O(^3^P) and O(^1^D) consume the CH_4_, leading it to build up more slowly with the least conservative prescription.

As a direct product of CO_2_ photolysis, the increased CO that is generated with the least conservative CO_2_ cross-section prescription is readily apparent in Figure 4. In this figure we see large differences in the resulting surface CO abundance depending on the CO_2_ cross-section prescription. Because of its high stellar flux overall, these differences are greatest for the F-type host star at the lowest CH_4_ flux, where there is over four orders of magnitude more surface CO modeled with the least conservative cross sections than with the most conservative.

The largest relative cross-section-dependent differences are seen with the surface O_2_ volume mixing ratios, as seen in Figure 5. Here, we see that the source of variation in predicted surface O_2_ is almost exclusive to the models run using the least conservative CO_2_ cross sections, which predict over eight orders of magnitude more surface O_2_ at low CH_4_ fluxes for TRAPPIST-1 as the host star. There are two exceptions to this: the first, with Sigma Boötis as the host star, where there are almost two orders of magnitude difference between the recommended and most conservative prescriptions. However, this difference occurs at very low O_2_ mixing ratios (e.g., 2.8 × 10^−14^ predicted with the most conservative prescription, versus 1.0 × 10^−12^ predicted with the recommended prescription), thus we would not expect a corresponding difference in the spectral observables of either O_2_ or O_3_. The other exception is with GJ 876 as the host star, which demonstrates a stepwise decrease in surface O_2_ at a CH_4_ flux of around 10^9^ molecules cm^−2^ s^−1^ regardless of the cross-section prescription. This is caused by variation in the threshold by which the CH_4_ collapses the O_2_ levels, and is particularly sensitive to input parameters such as the CH_4_ flux and cross sections. This strong sensitivity of O_2_ to reductant fluxes is commonly seen for late-type host stars, and is ultimately traced to their high far-UV/near-UV ratios (C. Harman et al. 2015; P. Barth et al. 2024).

Appendix B shows an alternative visualization of these trace gas abundance differences at specific CH_4_ fluxes of 10^8^, 10^9^, 10^10^, and 10^11^ molecules cm^−2^ s^−1^, for the F-, G-, and K-type host stars, as well as the M5.5eV host star in Figures 16, 17, and 18 in Appendix B.

#### 3.1.2. CO_2_ Surface Mixing Ratio Sensitivity Tests

**Figure 6.**
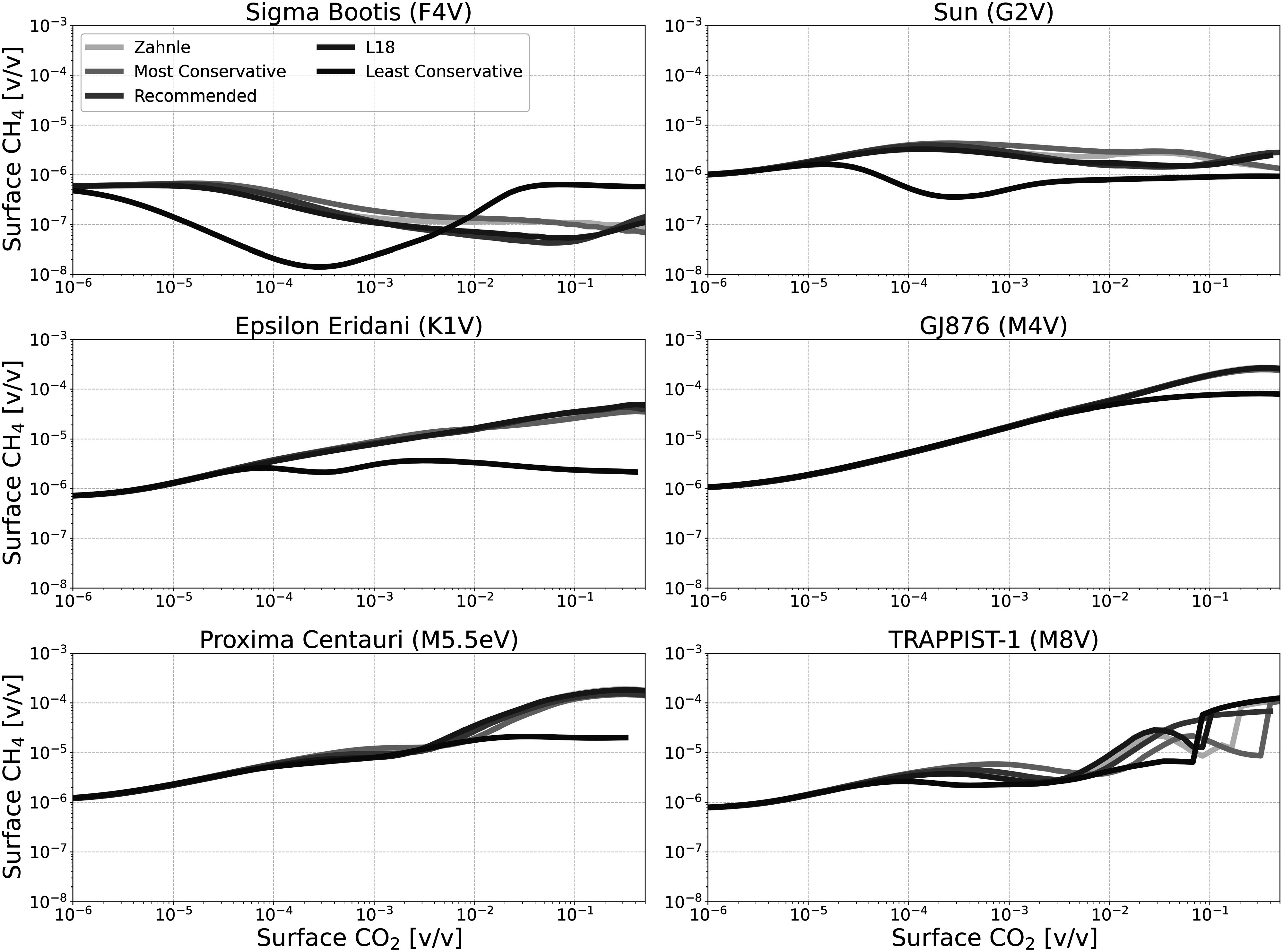
CO_2_ cross-section sensitivity test: surface CH_4_ vs. surface CO_2_ for anoxic habitable planets orbiting FGKM-type host stars.

**Figure 7.**
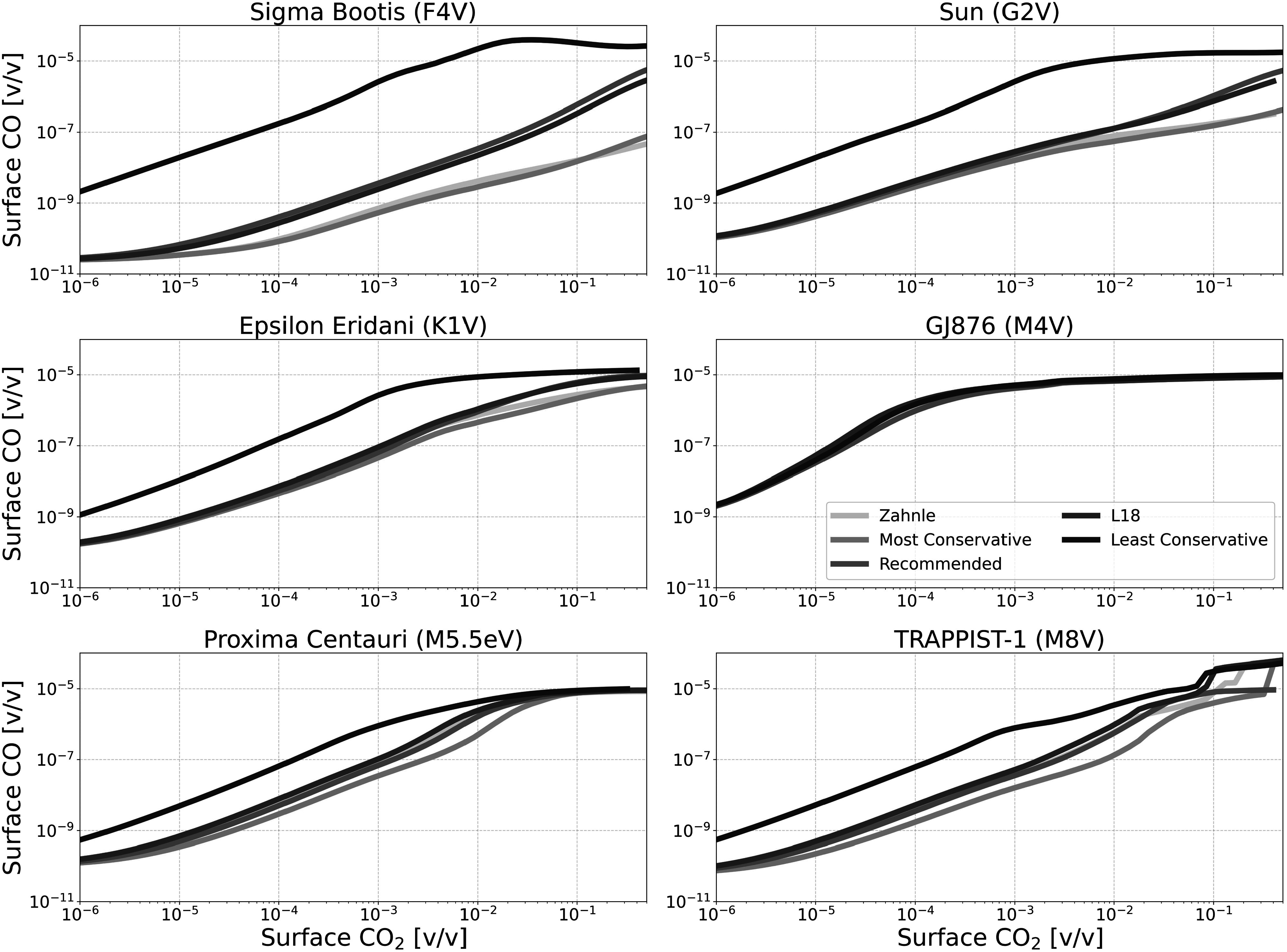
CO_2_ cross-section sensitivity test: surface CO vs. surface CO_2_ for anoxic habitable planets orbiting FGKM-type host stars.

**Figure 8.**
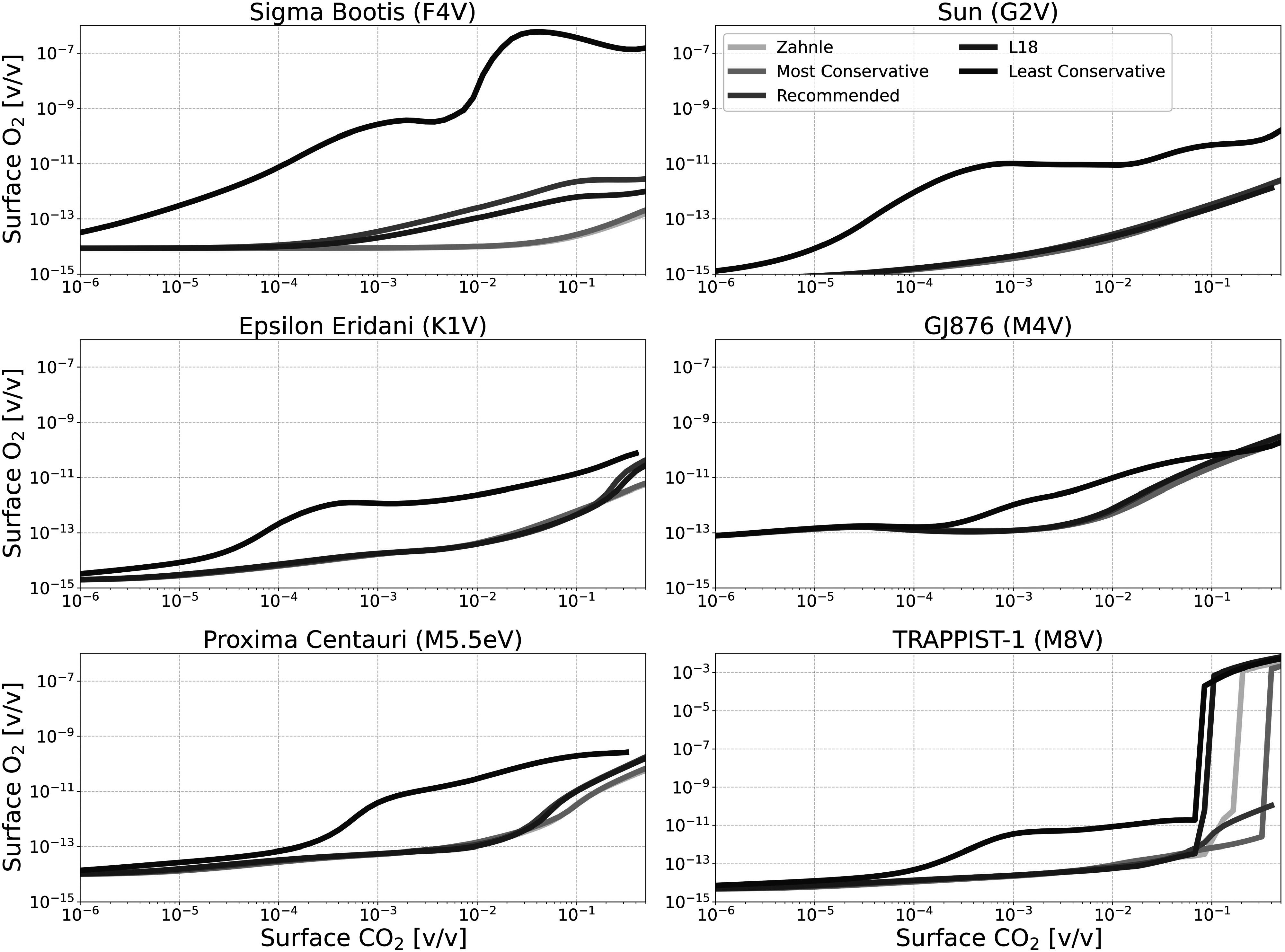
CO_2_ cross-section sensitivity test: surface O_2_ vs. surface CO_2_ for anoxic habitable planets orbiting FGKM-type host stars.

Figures 6, 7, and 8 show the impact of the various CO_2_ cross-section prescriptions on surface CH_4_, CO, and O_2_ as a function of CO_2_ surface mixing ratio, respectively. Here, we can see that the predicted trace gas abundances do not vary greatly between CO_2_ cross-section prescriptions, though where differences exist they are mostly exhibited by models run using the least conservative prescription.

 Appendix B shows an alternative visualization of these trace gas abundance differences at specific CO_2_ volume mixing ratios of fluxes of 10^−6^, 10^−4^, 10^−2^, and 3 × 10^−1^, for the F-, G-, and K-type host stars, as well as the M5.5eV host star in Figures 19, 20, and 21 in Appendix B.

### 3.2. Spectral Sensitivity

**Figure 9.**
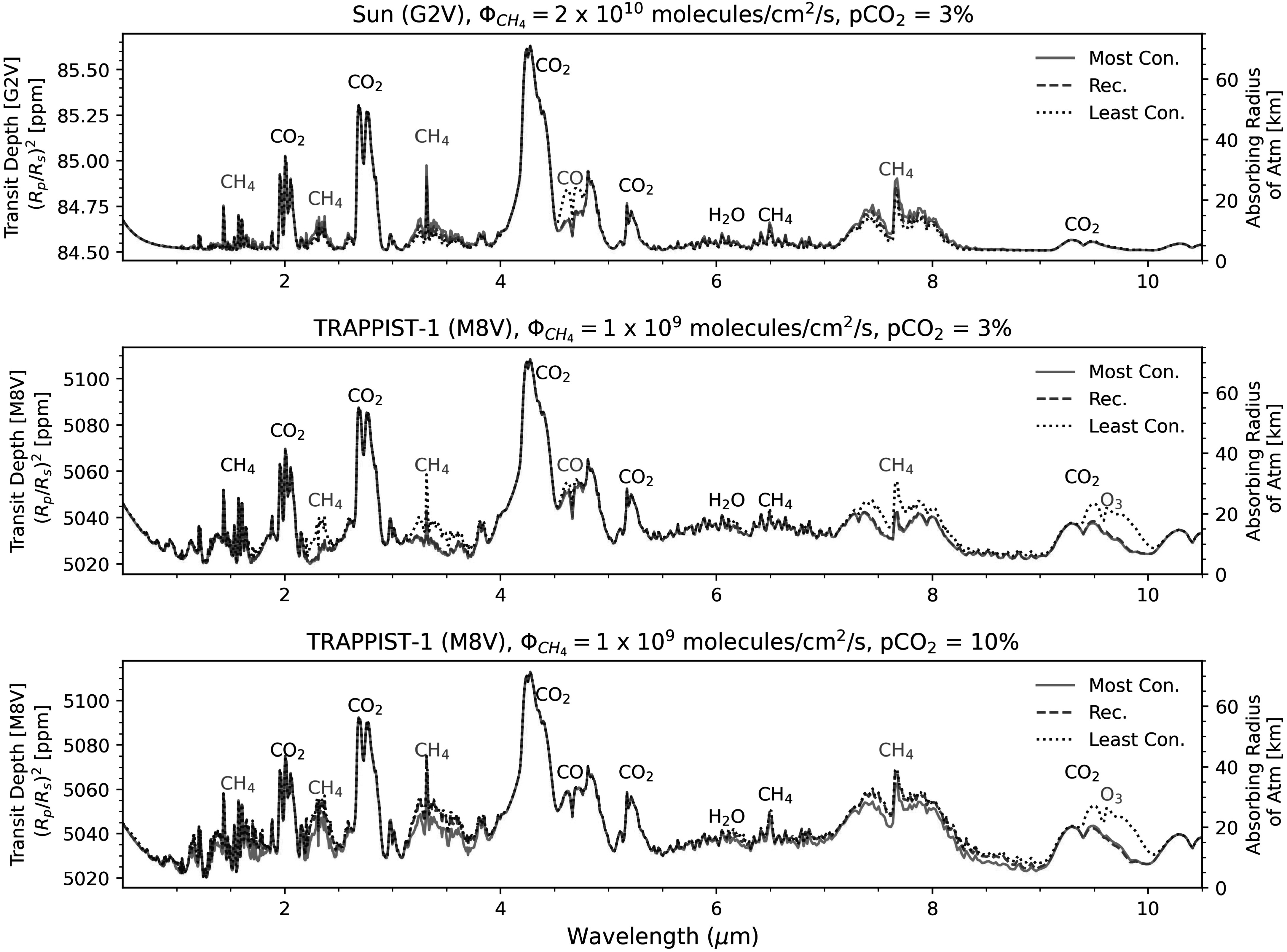
Comparison of transmission spectra between the recommended, most conservative, and least conservative CO_2_ prescriptions, for three different scenarios. The top panel shows transmission spectra for a planet orbiting the Sun, with a CH_4_ flux = 2 × 10^10^ molecules cm^−2^ s^−1^, with a surface CO_2_ volume mixing ratio of 3%. The middle and bottom panels show the transmission spectra for a planet orbiting TRAPPIST-1 with a CH_4_ flux = 1 × 10^9^ molecules cm^−2^ s^−1^; the middle also has a surface CO_2_ volume mixing ratio of 3%, while the bottom panel has a surface CO_2_ volume mixing ratio of 10%. Features that are shared between spectra are labeled in black; features that differ depending on the cross-section prescription are labeled in red.

**Figure 10.**
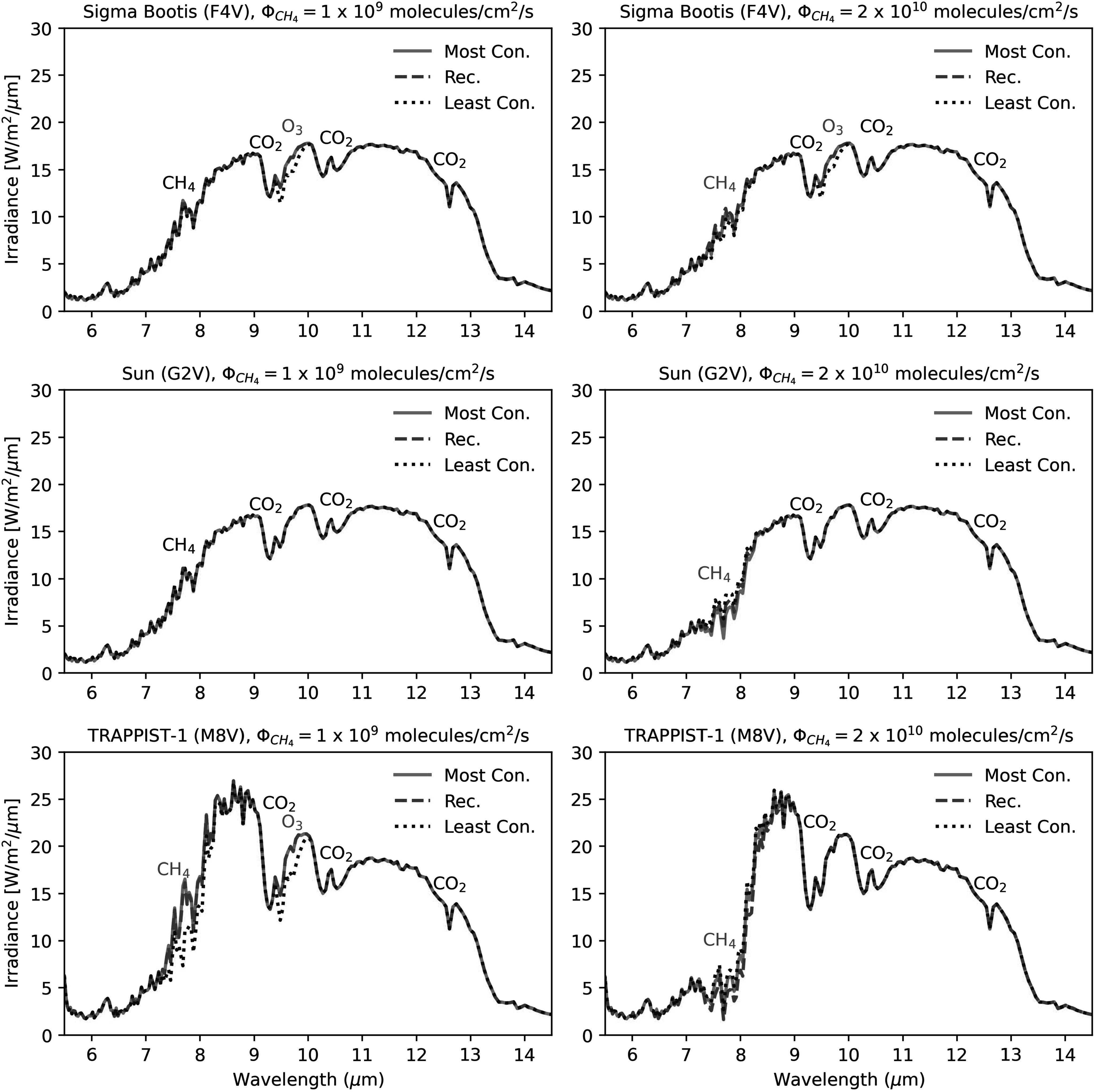
Comparison of emission spectra between the recommended, most conservative, and least conservative CO_2_ prescriptions, for two different CH_4_ fluxes (panels on the left are modeled with a CH_4_ flux = 1 × 10^9^ molecules cm^−2^ s^−1^, those on the right with a CH_4_ flux = 2 × 10^10^ molecules cm^−2^ s^−1^) with (from top to bottom) Sigma Boötis, the Sun, and TRAPPIST-1 as the host star. Features that are shared between spectra are labeled in black; features that differ depending on the cross-section prescription are labeled in red.

**Figure 11.**
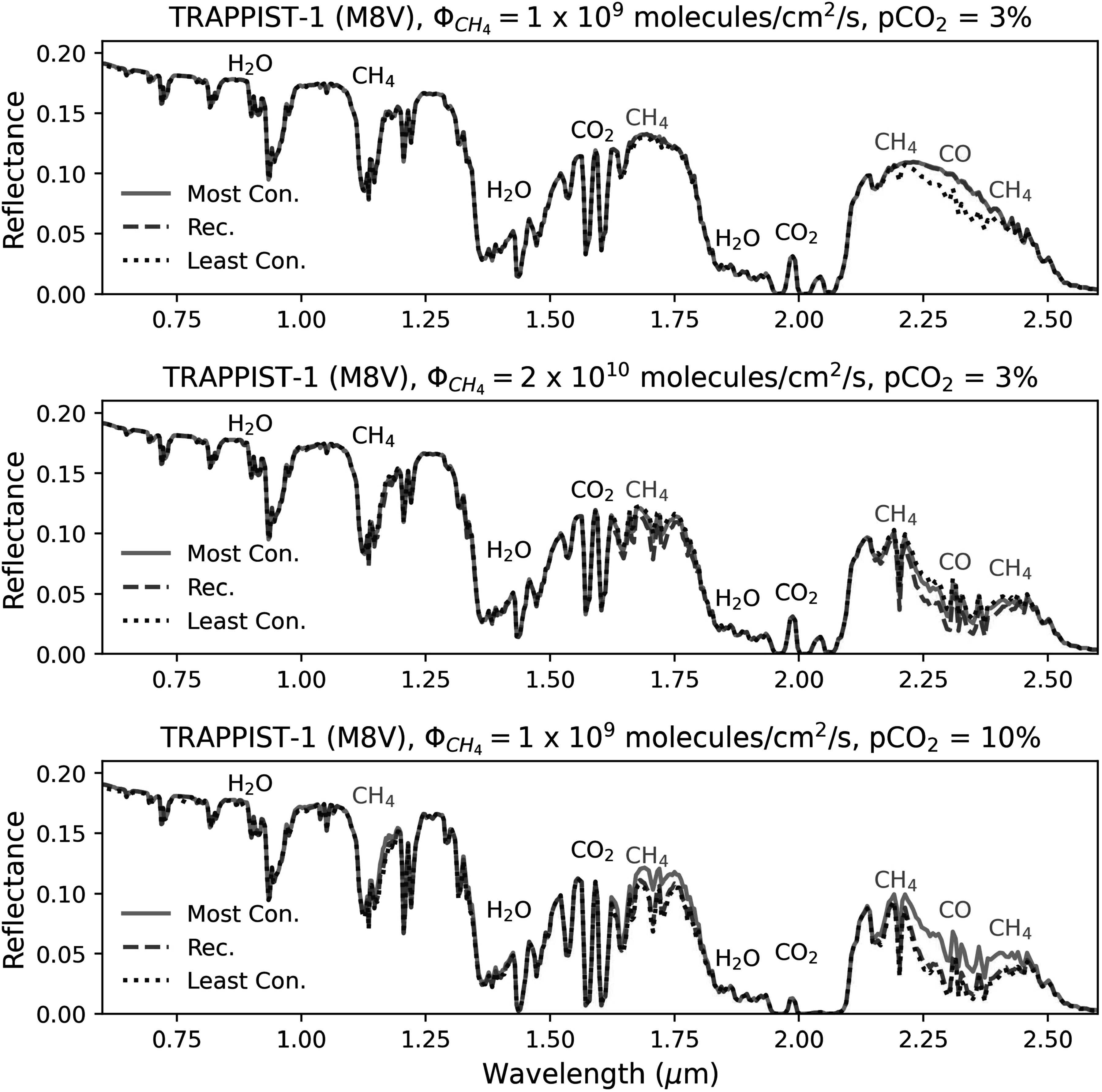
Comparison of reflection spectra between recommended, most conservative, and least conservative CO_2_ prescriptions, for TRAPPIST-1 as a host star. The top panel is modeled with a CH_4_ flux = 1 × 10^9^ molecules cm^−2^ s^−1^ and a surface CO_2_ volume mixing ratio of 3%, the middle panel is modeled with a CH_4_ flux = 2 × 10^10^ molecules cm^−2^ s^−1^ and a surface CO_2_ volume mixing ratio of 3%, and the bottom panel is modeled with a CH_4_ flux = 1 × 10^9^ molecules cm^−2^ s^−1^ and a surface CO_2_ volume mixing ratio of 10%. Features that are shared between spectra are labeled in black; features that differ depending on the cross-section prescription are labeled in red.

Depending on the choice of CO_2_ cross-section prescription, model predictions of atmospheric trace gas abundances can vary substantially, but that does not necessarily mean they will induce observable variations in planetary spectra. To demonstrate how these differing predictions may result in differences in the predicted observables, we model the transmission, emission, and reflected light spectra for various combinations of host star, CH_4_ flux, and CO_2_ mixing ratio, for models run using the most conservative, least conservative, and recommended CO_2_ cross-section prescriptions. These spectra are shown in Figures 9, 10, and 11, respectively. Spectral features shared between spectra are labeled in black text, and spectral features that differ depending on the cross-section prescription are labeled in red text. In all scenarios, the spectra were generated with an assumed cloud coverage of 50% clear sky, 25% cirrus clouds, and 25% stratus clouds, as in G. Arney et al. (2016). Planetary parameters used to model the given scenarios are provided in Table 2.

In Figure 9, we show the transmission spectra for three different scenarios. The top panel is modeled using the Sun as a host star with a planetary CH_4_ flux = 2 × 10^10^ molecules cm^−2^ s^−1^ and an atmospheric CO_2_ surface volume mixing ratio of 3%. This scenario corresponds to the example profile plot shown in the middle panel of Figure 2, and we can see that the greater levels of CO and lower levels of CH_4_ modeled using the least conservative CO_2_ cross sections lead to a larger CO feature around 4.6 *μ*m and smaller CH_4_ features around 1.7, 2.4, 3.3, and 7.6 *μ*m. In the middle panel of Figure 9, we show transmission spectra modeled using TRAPPIST-1 as the host star, with planetary parameters emulating TRAPPIST-1 e, a CH_4_ flux = 1 × 10^9^ molecules cm^−2^ s^−1^ and a CO_2_ surface volume mixing ratio = 3%. Here we see that the increased surface CH_4_ predicted using the least conservative prescription leads to larger spectral features due to CH_4_, particularly the CH_4_ peak around 3.3 *μ*m. In addition to the CH_4_ features, the CO around 4.6 *μ*m is slightly more prominent with the least conservative prescription, and, most notably, the spectrum modeled with the least conservative prescription shows an O_3_ feature around 9.6 *μ*m that is not seen in the other spectra of this panel. The bottom panel of Figure 9 again uses TRAPPIST-1 as the host star with a CH_4_ flux = 1 × 10^9^ molecules cm^−2^ s^−1^, now with a CO_2_ surface volume mixing ratio = 10%. The decreased CH_4_ predicted with the most conservative prescription leads to smaller CH_4_ spectral features at 1.7, 2.4, 3.3, and 7.6 *μ*m, and we still see an O_3_ feature around 9.6 *μ*m with the spectrum modeled using the least conservative prescription that is not seen with the spectra modeled using either the most conservative or the recommended prescriptions. Ultimately, the use of either the most conservative or least conservative prescription in models results in spectral differences from those using the recommended prescription; however, the predictions from the most conservative and the recommended prescriptions are most similar. In particular, predicted O_3_ and CO features are always larger when using the least conservative CO_2_ dissociation prescriptions.

Figure 10 shows six emission spectra scenarios; on the left, we show emission spectra for (from top to bottom:) Sigma Boötis, the Sun, and TRAPPIST-1 as the host star, for a CH_4_ flux = 1 × 10^9^ molecules cm^−2^ s^−1^ and a CO_2_ surface volume mixing ratio = 3%, and on the right, the three spectra correspond to the example profile plots shown in Figure 2. Overall, we do not see as great a cross-section-dependent difference in these spectra, with the exception of the bottom left panel where we see that the spectrum modeled using the least conservative prescription shows a deeper CH_4_ feature around 7 *μ*m, as well as an O_3_ feature that is not present in the spectra that use the most conservative or recommended prescription.

In Figure 11, we show three scenarios for reflection spectra using TRAPPIST-1 as the host star. The top panel shows spectra modeled using a CH_4_ flux = 1 × 10^9^ molecules cm^−2^ s^−1^ with the surface CO_2_ = 3%, the middle panel shows spectra modeled using a CH_4_ flux = 2 × 10^10^ molecules cm^−2^ s^−1^ with the surface CO_2_ = 3%, and the bottom panel shows spectra modeled using a CH_4_ flux = 1 × 10^9^ molecules cm^−2^ s^−1^ with the surface CO_2_ = 10%. All three scenarios show differences around 1.7, 2.2, and 2.4 *μ*m due to CH_4_, as well as a difference due to CO around 2.3 *μ*m.

In general, we find that spectra generated when using the least conservative CO_2_ cross-section prescription differ the most compared to the spectra generated when using the most conservative and recommended cross-section prescriptions.

### 3.3. Revisiting the H_2_O Cross-section Sensitivity Tests

**Figure 12.**
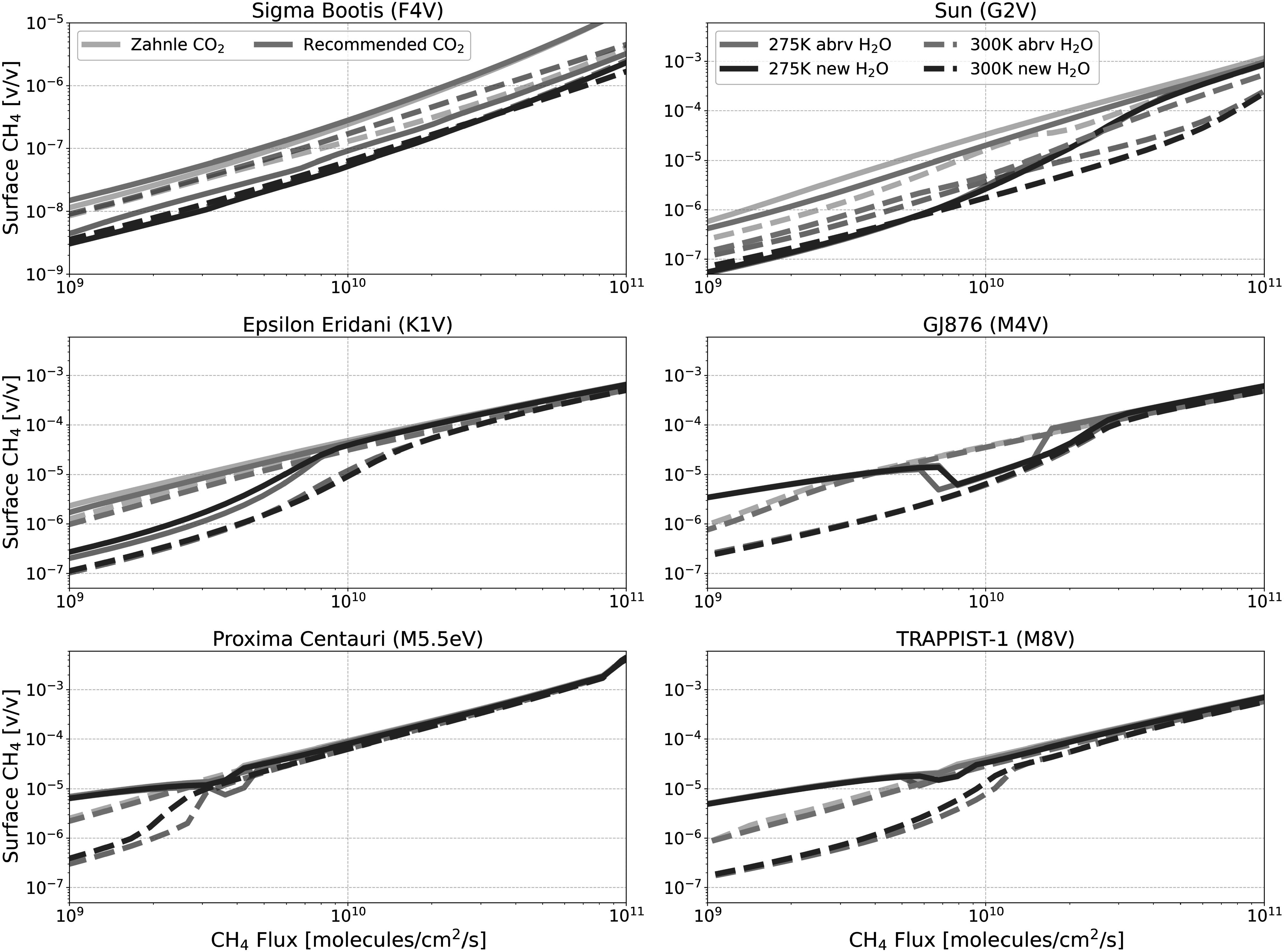
Comparison of H_2_O cross-section sensitivity tests with old and recommended CO_2_ cross sections; surface CH_4_ vs. CH_4_ flux for anoxic habitable planets orbiting FGKM-type host stars.

**Figure 13.**
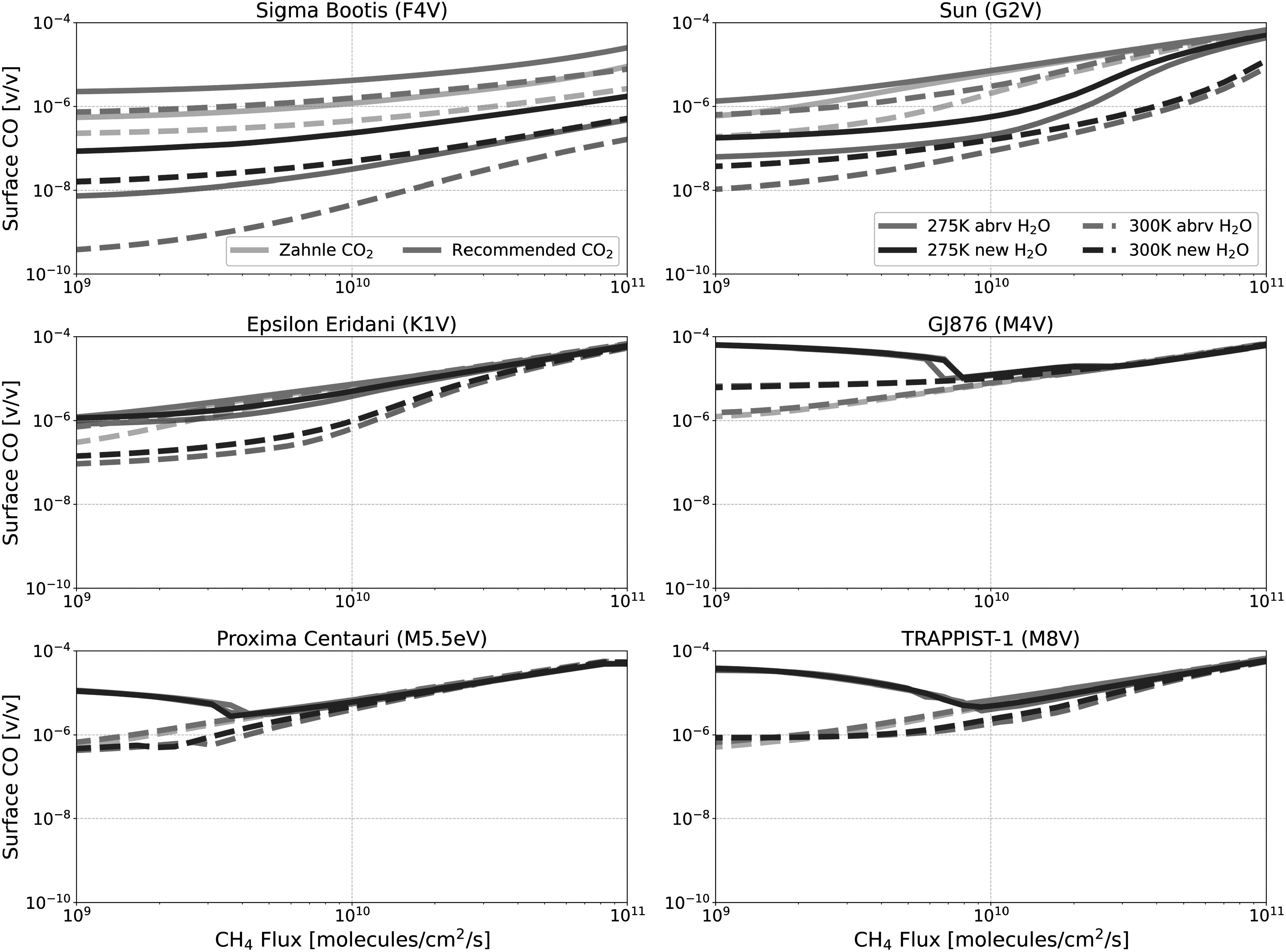
Comparison of H_2_O cross-section sensitivity tests with old and recommended CO_2_ cross sections; surface CO vs. CH_4_ flux for anoxic habitable planets orbiting FGKM-type host stars.

**Figure 14.**
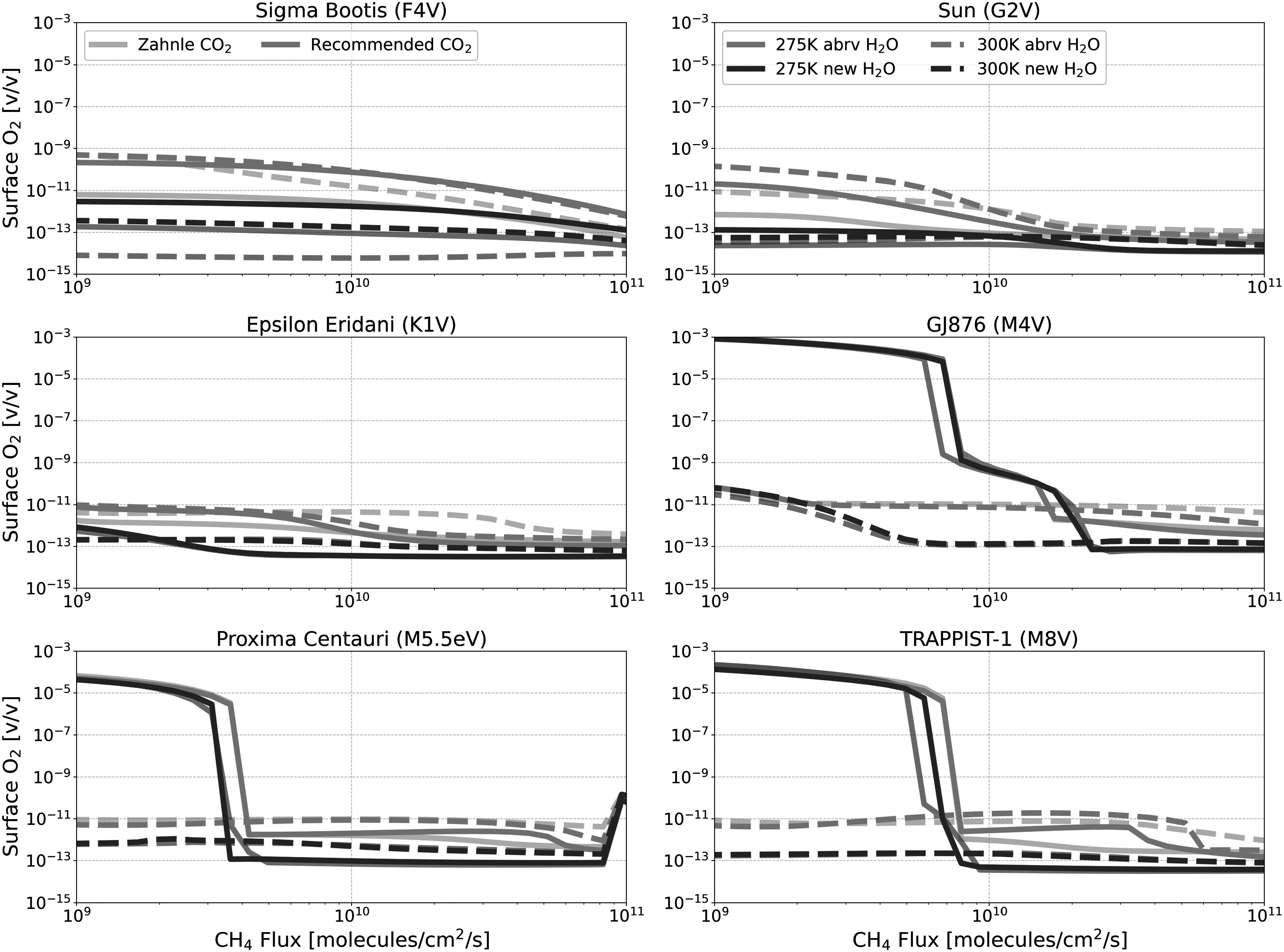
Comparison of H_2_O cross-section sensitivity tests with old and recommended CO_2_ cross sections; surface O_2_ vs. CH_4_ flux for anoxic habitable planets orbiting FGKM-type host stars.

We have revisited the H_2_O cross-section sensitivity tests conducted by W. Broussard et al. (2024) with the newly recommended CO_2_ cross sections, to see how these previous results are impacted. Specifically, we show the impact on the tests conducted using the new H_2_O cross sections from S. Ranjan et al. (2020), and the abbreviated versions of these H_2_O cross sections, which use a cutoff of 200 nm. Figures 12, 13, and 14 show the impact on the surface CH_4_, CO, and O_2_ volume mixing ratios respectively. Broadly, the results from W. Broussard et al. (2024) remain unchanged; terminating the H_2_O cross sections at 200 nm results in less H_2_O photolysis, thus less OH is produced and trace gases can build up to higher levels, with the cross-section-dependent differences being more pronounced for the FGK-type host stars and negligible for the M-type host stars. By incorporating the recommended CO_2_ cross sections, we see that the magnitude of the differences has modestly decreased. For example, when using the Zahnle CO_2_ cross sections to model a planet orbiting the Sun with the 275 K surface temperature regime and a CH_4_ surface flux of 1 × 10^10^ molecules cm^−2^ s^−1^, the abbreviated H_2_O cross sections predict a surface CH_4_ abundance that is 10 times greater than the surface CH_4_ abundance predicted when using the new H_2_O cross sections. When modeling this same scenario with the recommended CO_2_ cross sections, the surface CH_4_ abundance is only 7 times greater when modeled using the abbreviated H_2_O cross sections than with the new H_2_O cross sections.

**Figure 15.**
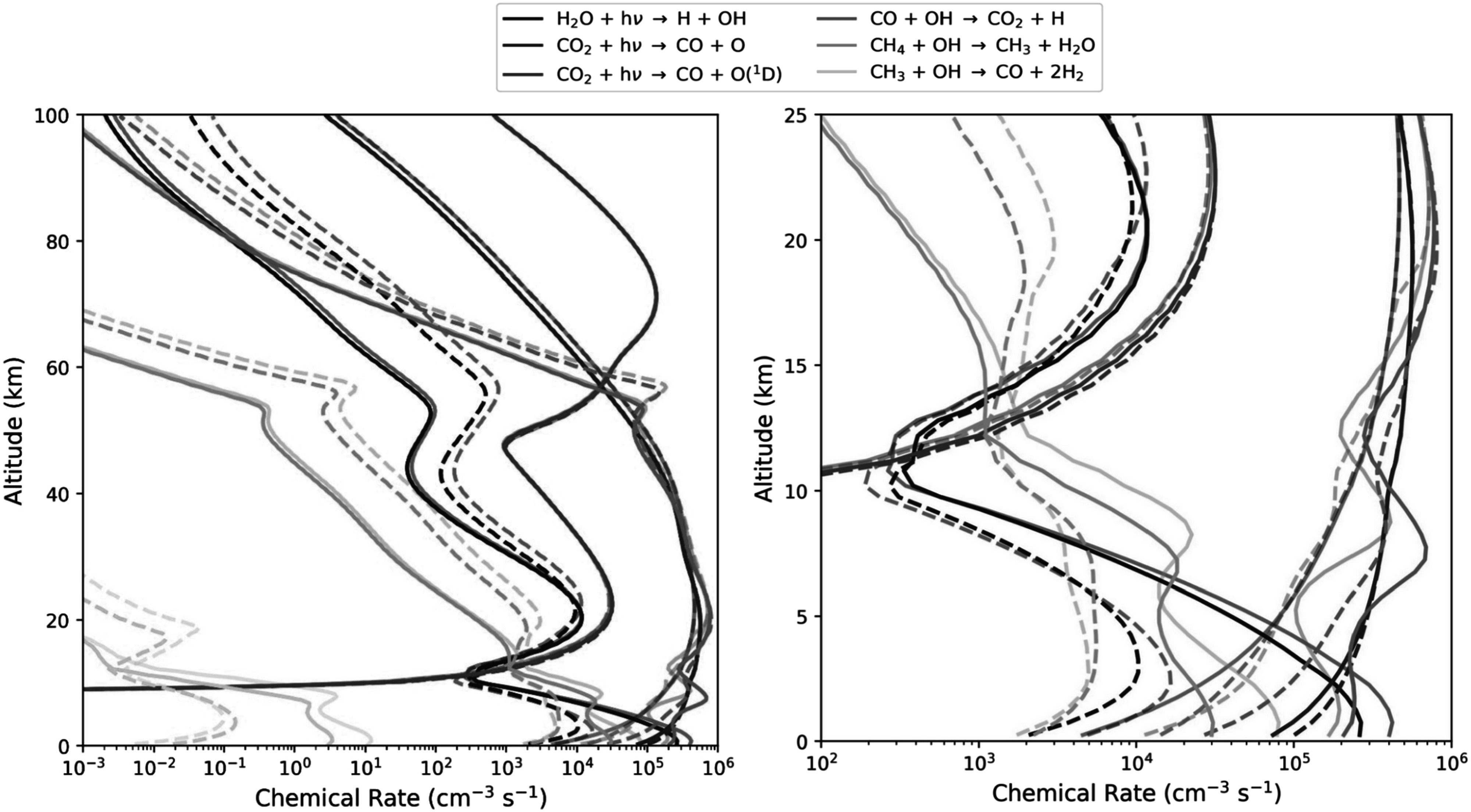
Comparison of the reaction rates from the H_2_O cross-section sensitivity tests with old and recommended CO_2_ cross sections for a habitable anoxic planet orbiting the Sun, for a CH_4_ flux of 1.1 × 10^10^ molecules cm^−2^ s^−1^ and a 275 K surface temperature regime. Solid lines are reaction rates modeled using the new H_2_O cross sections, dashed lines are reaction rates modeled using the abbreviated H_2_O cross sections; results from W. Broussard et al. (2024) are plotted with 50% opacity, and updated results using the recommended CO_2_ cross sections are plotted with 100% opacity. On the left, reaction rates up to 100 km are shown; the panel on the right shows the reaction rates up to 25 km.

**Figure 16.**
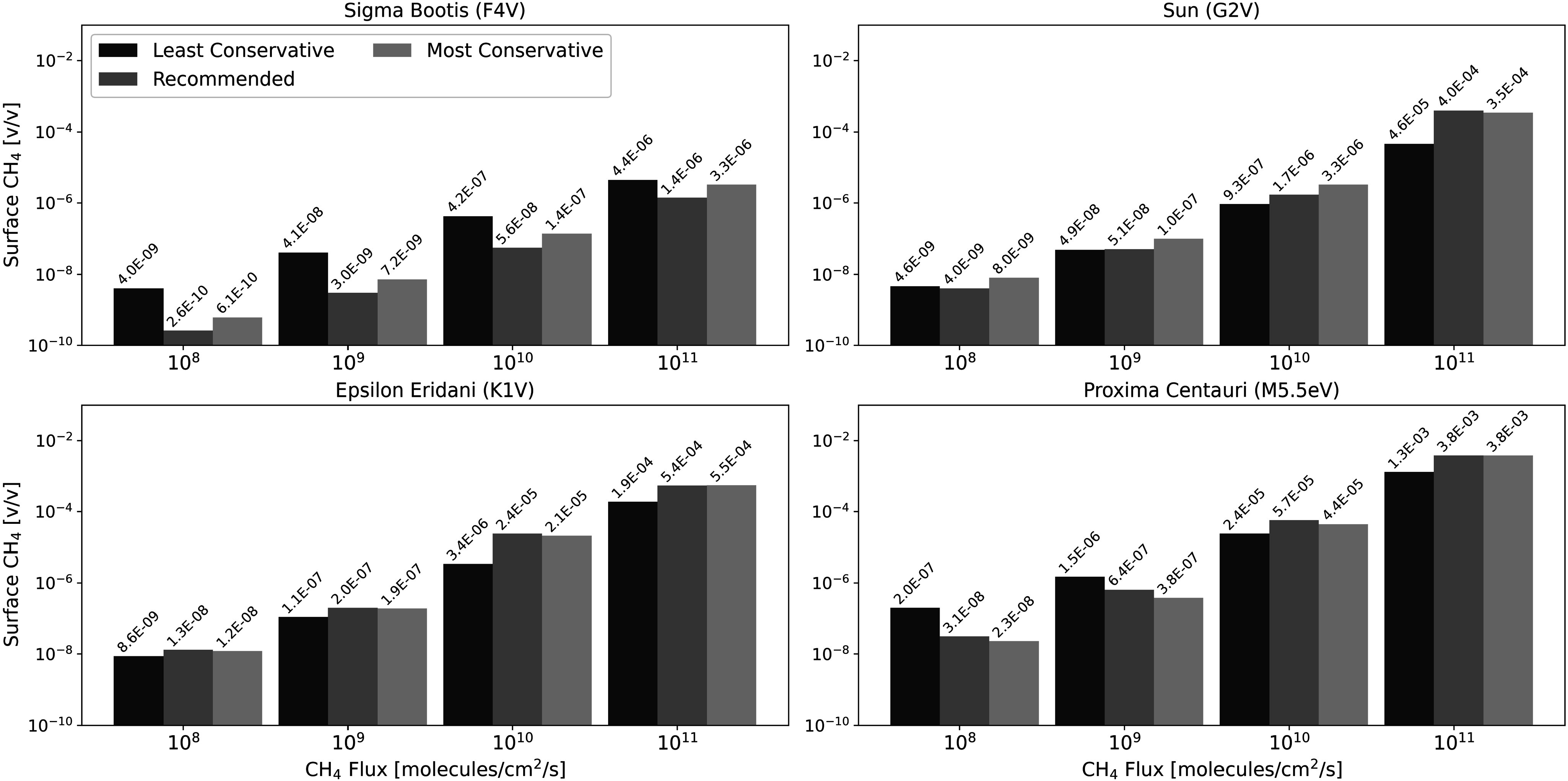
Quantitative difference between CH_4_ surface mixing ratios for CH_4_ surface fluxes of 10^8^, 10^9^, 10^10^, and 10^11^ molecules cm^−2^ s^−1^, for anoxic habitable planets modeled using Sigma Boötis, the Sun, Epsilon Eridani, and Proxima Centauri as the host star.

**Figure 17.**
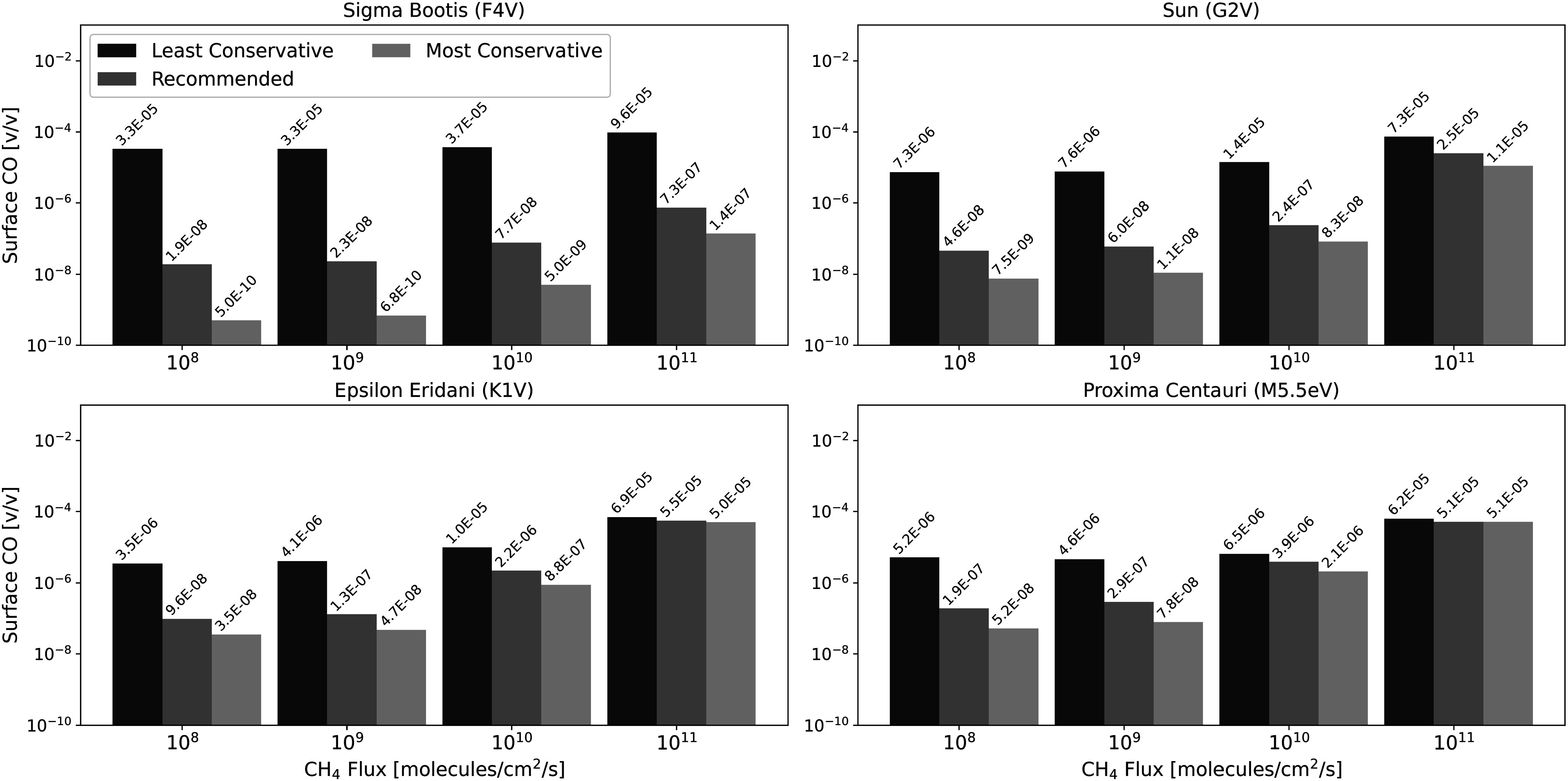
Quantitative difference between CO surface mixing ratios for CH_4_ surface fluxes of 10^8^, 10^9^, 10^10^, and 10^11^ molecules cm^−2^ s^−1^, for anoxic habitable planets modeled using Sigma Boötis, the Sun, Epsilon Eridani, and Proxima Centauri as the host star.

**Figure 18.**
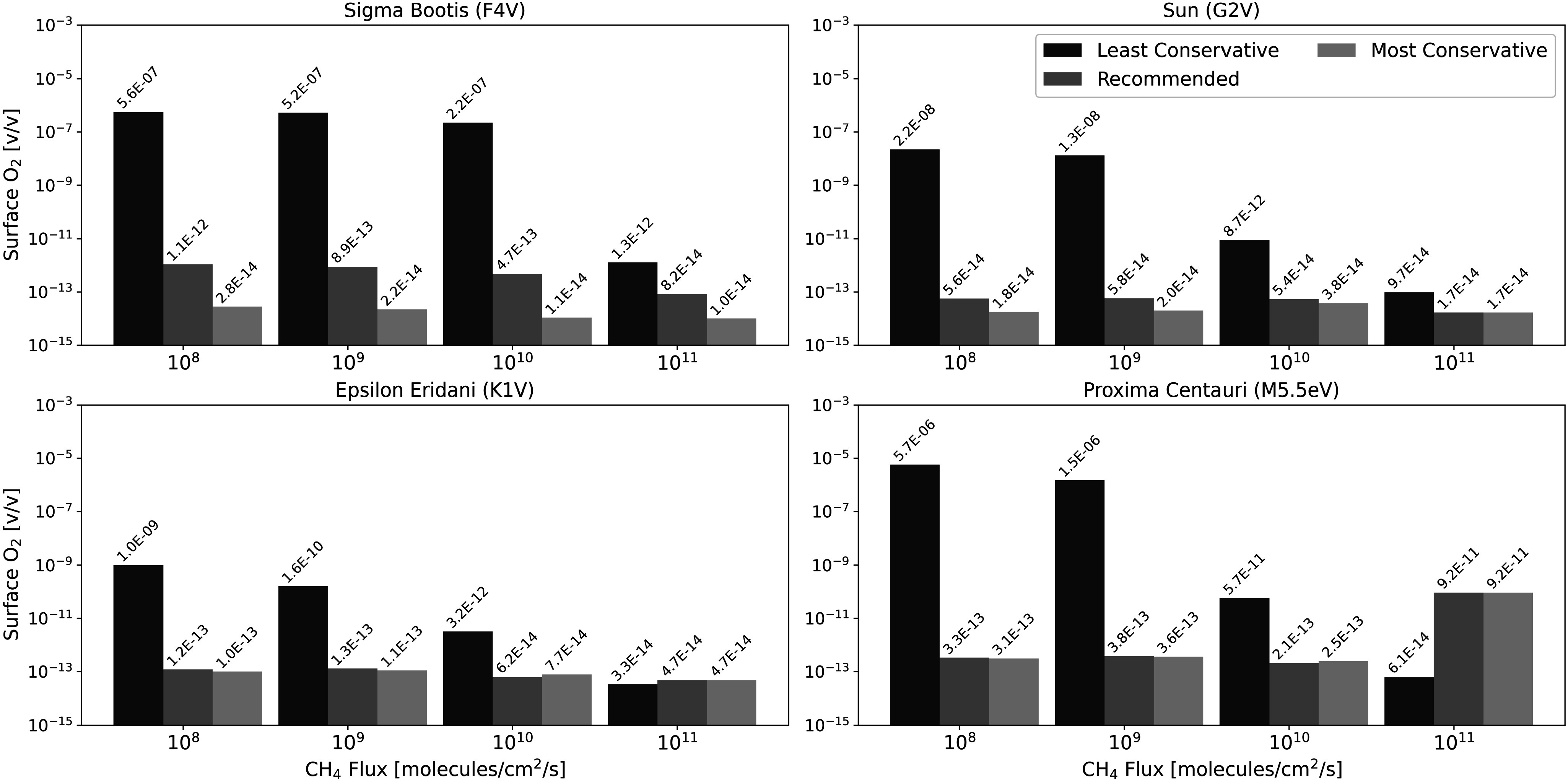
Quantitative difference between O_2_ surface mixing ratios for CH_4_ surface fluxes of 10^8^, 10^9^, 10^10^, and 10^11^ molecules cm^−2^ s^−1^, for anoxic habitable planets modeled using Sigma Boötis, the Sun, Epsilon Eridani, and Proxima Centauri as the host star.

**Figure 19.**
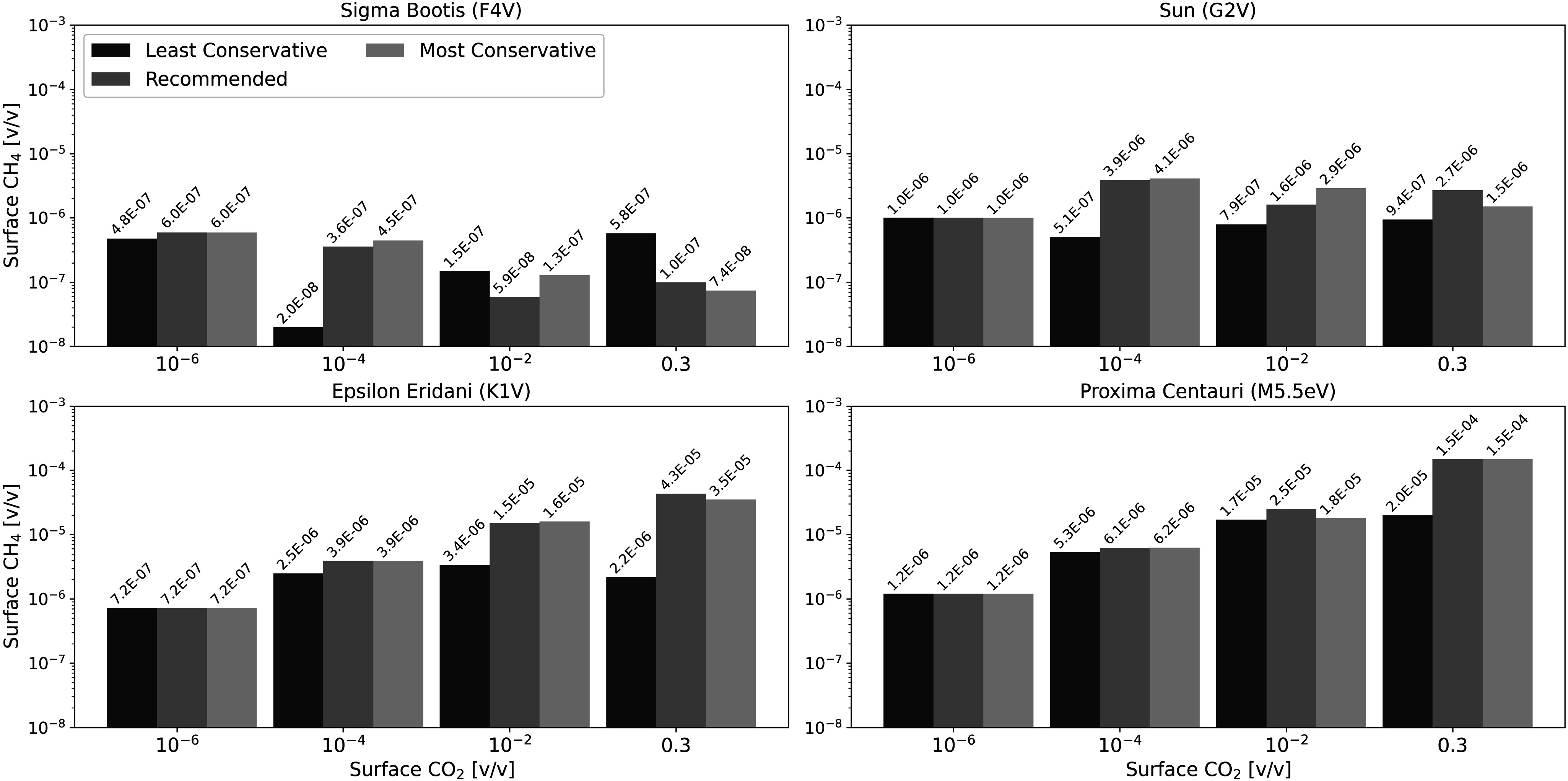
Quantitative difference between CH_4_ surface mixing ratios for CO_2_ surface mixing ratios of 10^−6^, 10^−4^, 10^−2^, and 0.3 (v/v), for anoxic habitable planets modeled using Sigma Boötis, the Sun, Epsilon Eridani, and Proxima Centauri as the host star.

**Figure 20.**
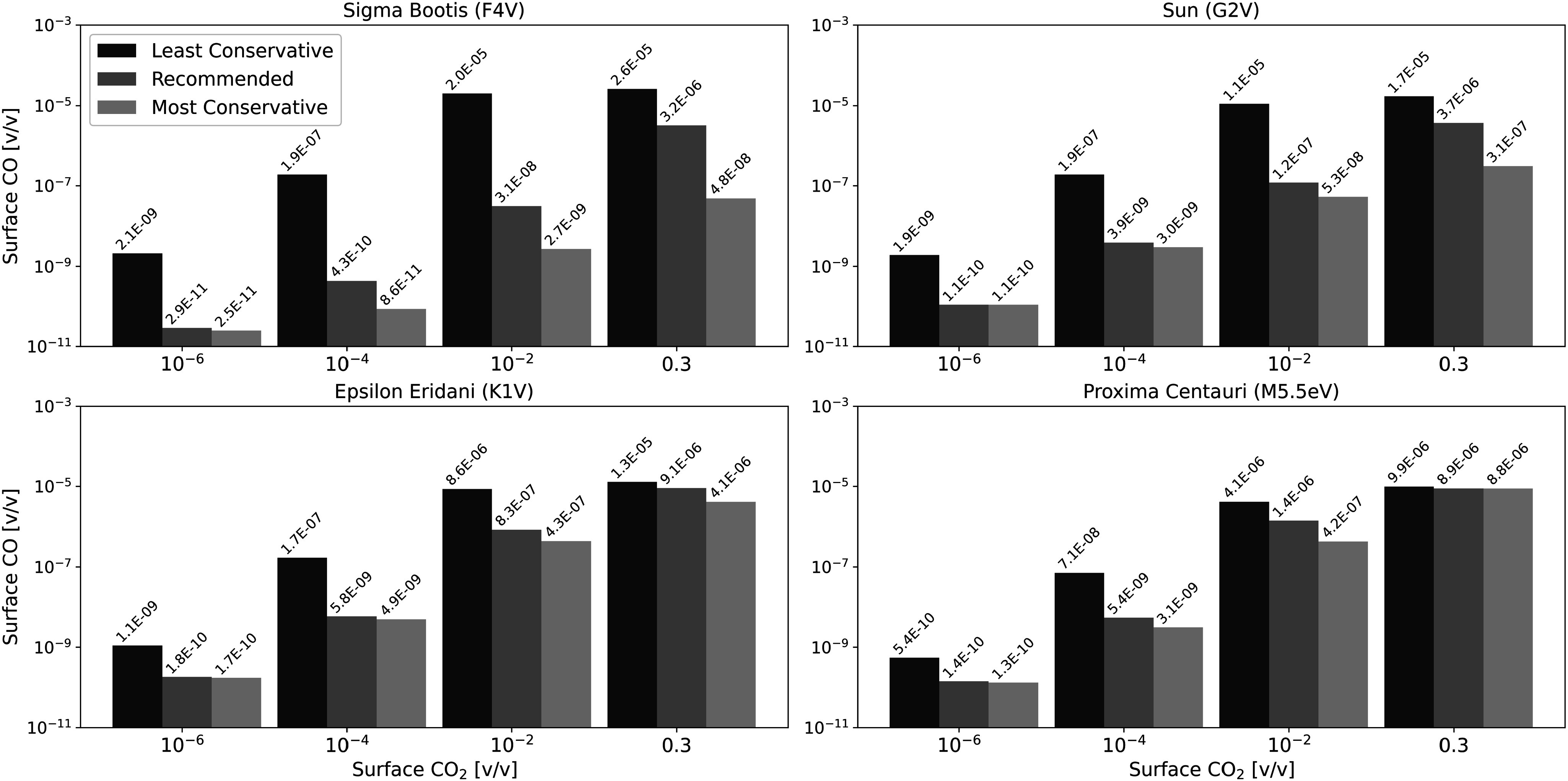
Quantitative difference between CO surface mixing ratios for CO_2_ surface mixing ratios of 10^−6^, 10^−4^, 10^−2^, and 0.3 (v/v), for anoxic habitable planets modeled using Sigma Boötis, the Sun, Epsilon Eridani, and Proxima Centauri as the host star.

**Figure 21.**
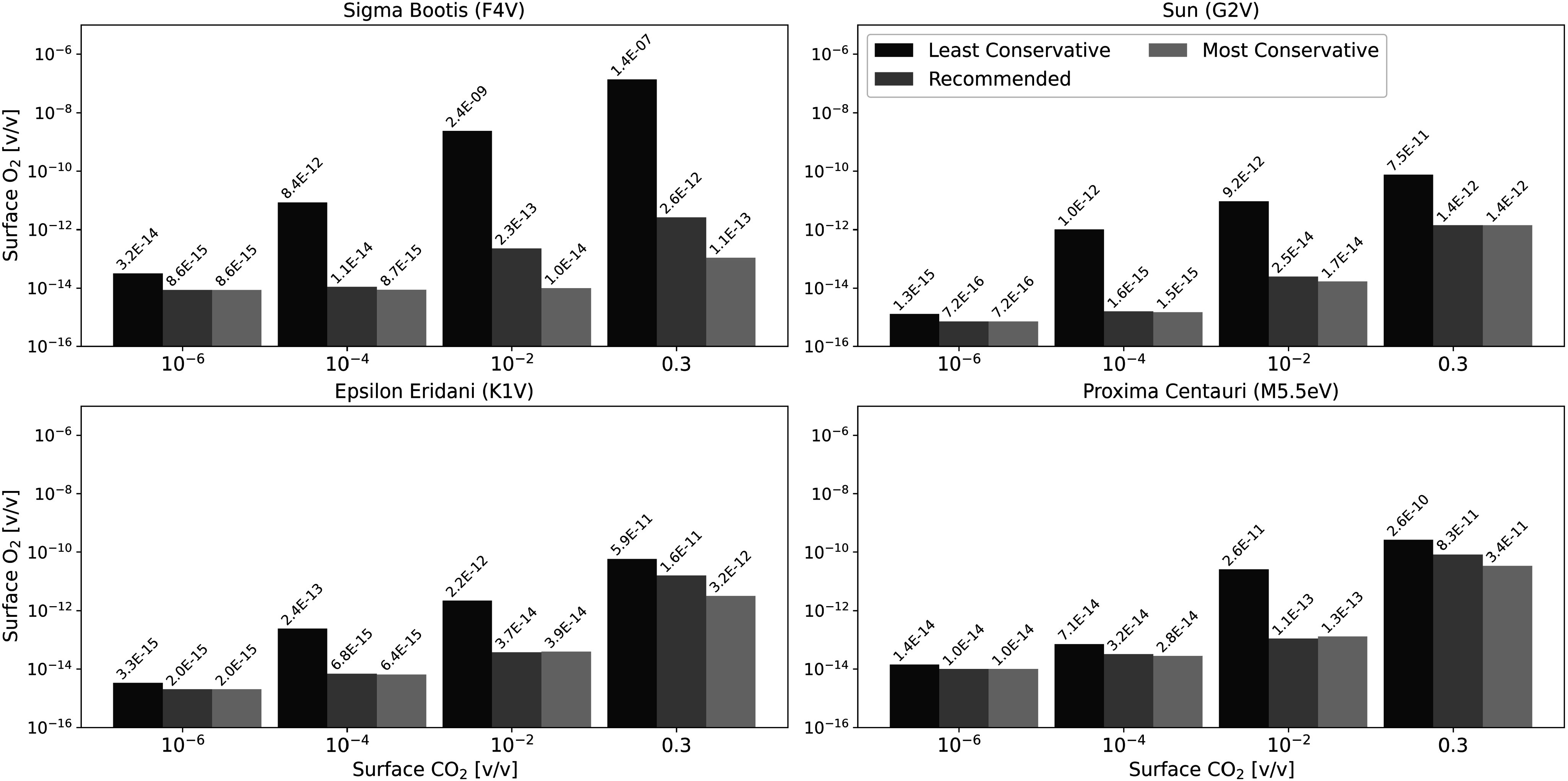
Quantitative difference between O_2_ surface mixing ratios for CO_2_ surface mixing ratios of 10^−6^, 10^−4^, 10^−2^, and 0.3 (v/v), for anoxic habitable planets modeled using Sigma Boötis, the Sun, Epsilon Eridani, and Proxima Centauri as the host star.

Figure 15 shows how key chemical reaction rates have changed with the recommended CO_2_ cross sections for a habitable anoxic planet orbiting the Sun, for the 275 K surface temperature regime with a CH_4_ flux of 1.1 × 10^10^ molecules cm^−2^ s^−1^. As expected, with the recommended CO_2_ cross sections extending further beyond 200 nm, more CO_2_ photolysis occurs, depleting actinic photons available for H_2_O photolysis in the troposphere. However, above the troposphere the amount of CO_2_ photolysis actually decreases with the recommended CO_2_ cross sections. This is likely because while the recommended CO_2_ cross sections include temperature-dependent calculations, the Zahnle CO_2_ cross sections do not. Since temperature decreases above the troposphere, the recommended prescription utilizes the 195 K cross sections, which are smaller than the Zahnle cross sections at wavelengths shorter than around 190 nm.

## 4. Discussion

The TRAPPIST-1 planetary system represents a prime opportunity for demonstrating JWST’s capability to detect and characterize secondary atmospheres on planets orbiting mid-to-late M-type stars (TRAPPIST-1 JWST Community Initiative et al. 2024). There is currently a strong community focus on studying the TRAPPIST-1 planets, and JWST transmission spectra of these planets will reveal consequential insights into the ability of terrestrial planets orbiting M-type stars to retain their atmospheres (TRAPPIST-1 JWST Community Initiative et al. 2024). As we can see from the transmission spectra modeled in Figure 9, the choice of CO_2_ cross-section prescription can have a consequential impact on the resulting transmission spectrum. Ruling out our least conservative, highest opacity CO_2_ prescription could meaningfully affect the interpretation of potential JWST transmission spectra.

A. H. M. J. Triaud et al. (2024) propose the depletion of atmospheric carbon, relative to other planets in the same system, as a potential biosignature. This potential biosignature would be supported by the presence of O_3_, which could distinguish between a habitable planet and an inhabited one, assuming the O_3_ is an indirect product of oxygenic photosynthesis. However, we predict that O_3_ could appear on an abiotic habitable planet assuming our least conservative CO_2_ prescription, which enhances CO_2_ photolysis and abiotic O_2_/O_3_ production. This could also be problematic for interpreting observations of planets orbiting F-type stars, as the least conservative CO_2_ cross sections led to a feature of O_3_ in the emission spectrum for both the scenario of CH_4_ flux = 1 × 10^9^ molecules cm^−2^ s^−1^ and that of CH_4_ flux = 2 × 10^10^ molecules cm^−2^ s^−1^. In other words, this is a possible false positive for the proposed CH_4_–O_2_ disequilibrium biosignature (C. Sagan et al. 1993). However, future measurements that conclusively exclude the least conservative CO_2_ absorption cross sections presented here would preclude these challenging scenarios.

For emission spectra of planets orbiting Sun-like G-type stars with the upcoming HWO, the resulting spectra are more robust against the different predictions based on CO_2_ cross-section prescription. As shown in Figure 10, with the Sun as a host star and a surface CH_4_ flux of 10^9^ molecules cm^−2^ s^−1^ the resulting emission spectra show no cross-section-dependent differences. Even when increasing the surface CH_4_ flux to 2 × 10^10^ molecules cm^−2^ s^−1^, the resulting spectra show a maximum percentage difference of about 45% (corresponding to an absolute difference of ∼2 W m^−2^* μ*m^−1^) around 7.8 *μ*m.

This research is limited in that it models the impact of extended CO_2_ cross sections on the trace gas abundances of CH_4_, CO, and O_2_ in Archean Earth-like N_2_–CO_2_–H_2_O-dominated atmospheres. This scenario represents just one of the many different possible planetary archetypes we may observe for exoplanets. Our photochemical predictions of other atmospheric scenarios may be more or less impacted by the different CO_2_ cross-section extrapolations. In particular, the temperature-dependent behavior of the CO_2_ cross sections may lead to greater cross-section-dependent differences for other planetary scenarios. For example, an oxygen-rich planet with a stratospheric ozone layer will also have an increased stratospheric temperature. Because the least conservative CO_2_ cross sections are so much larger at higher temperatures, scenarios modeled using this prescription would exhibit even stronger temperature-dependent differences than those modeled with the other prescriptions. Overall, the best way to decrease the uncertainty of our forward and retrieval models for all planetary scenarios would be to obtain additional high-quality CO_2_ cross-section data at MUV wavelengths.

## 5. Conclusions

We have tested the impact of extended CO_2_ cross sections (>200 nm) on the atmospheric trace gas abundance of anoxic, temperate, terrestrial exoplanet atmospheres, over a range of CH_4_ fluxes and CO_2_ surface mixing ratios for planets orbiting FGKM-type stars. Overall, we can see up to several orders of magnitude in variation of certain trace gas abundances, depending on the cross-section prescription; however, this is with the caveat that the majority of the variation comes in when considering the least conservative prescription and much of this variation is at mixing ratios too small to be observable. If we were able to rule out the least conservative prescription, we would be able to eliminate a large source of the uncertainty in the resulting atmospheric trace gas abundances that could plausibly be spectrally detected. For example, demonstrating that ${\sigma }_{{{\mathrm{CO}}}_{2}}< 1\times 1{0}^{-24}$ cm^−2^ for *λ* = 210–220 nm would strongly falsify the “least conservative” prescription.

The results presented here do not change the conclusions of W. Broussard et al. (2024) in terms of the sensitivity of trace gas abundances in temperate anoxic atmospheres to extended H_2_O cross sections. Having accurate fundamental modeling inputs is essential for modeling the atmospheres of potentially habitable planets and for being able to correctly interpret observations of terrestrial exoplanets. Repeated measurements of CO_2_’s cross sections in the MUV at temperatures relevant for habitability—in particular, measurements that are precise enough to rule out the least conservative prescription presented here—will allow us to shed the largest amount of uncertainty and rule out the most incompatible model results.

Overall, this work emphasizes the urgent need for additional laboratory and ab initio studies on fundamental photochemical factors, such as absorption cross sections. Having precise model inputs is essential for interpreting exoplanet spectra, which may eventually reveal atmospheric chemical signatures of life.

## Appendix A Biotic Atmospheric Boundary Conditions

Table 3 lists the biotic atmospheric boundary conditions used for modeling results from the main text of this paper. These are the surface fluxes, surface volume mixing ratios, and dry deposition velocities where relevant.

**Table 3:** Atmospheric Species Boundary Conditions

Species	Surface Flux	Surface Mixing Ratio	Dry Deposition Velocity
	(molecules cm^−2^ s^−1^)	(v/v)	(cm s^−1^)
O(^3^P)	⋯	⋯	1
O_2_	⋯	⋯	0
H_2_O	⋯	⋯	0
H	⋯	⋯	1
OH	⋯	⋯	1
HO_2_	⋯	⋯	1
H_2_O_2_	⋯	⋯	2 × 10^−1^
H_2_	1 × 10^10^	⋯	2.4 × 10^−4^
CO	1 × 10^8^	⋯	1.2 × 10^−4^
HCO	⋯	⋯	1
H_2_CO	⋯	⋯	2 × 10^−1^
CH_4_	10^9^–10^11^	⋯	⋯
CH_3_	⋯	⋯	1
C_2_H_6_	⋯	⋯	0
NO	⋯	⋯	3 × 10^−4^
NO_2_	⋯	⋯	3 × 10^−3^
HNO	⋯	⋯	1
O_3_	⋯	⋯	7 × 10^−2^
HNO_3_	⋯	⋯	2 × 10^−1^
N	⋯	⋯	0
C_3_H_2_	⋯	⋯	0
C_3_H_3_	⋯	⋯	0
CH_3_C_2_H	⋯	⋯	0
CH_2_CCH_2_	⋯	⋯	0
C_3_H_5_	⋯	⋯	0
C_3_H_6_	⋯	⋯	0
C_3_H_7_	⋯	⋯	0
C_3_H_8_	⋯	⋯	0
C_2_H_4_OH	⋯	⋯	0
C_2_H_2_OH	⋯	⋯	0
C_2_H_5_	⋯	⋯	0
C_2_H_4_	⋯	⋯	0
CH	⋯	⋯	0
CH_3_O_2_	⋯	⋯	0
CH_3_O	⋯	⋯	0
CH_2_CO	⋯	⋯	0
CH_3_CO	⋯	⋯	0
CH_3_CHO	⋯	⋯	0
C_2_H_2_	⋯	⋯	0
CH_2_3	⋯	⋯	0
C_2_H	⋯	⋯	0
C_2_	⋯	⋯	0
C_2_H_3_	⋯	⋯	0
HCS	⋯	⋯	0
CS_2_	⋯	⋯	0
CS	⋯	⋯	0
OCS	⋯	⋯	0
S	⋯	⋯	0
HS	⋯	⋯	0
H_2_S	3.5 × 10^8^	⋯	2 × 10^−2^
SO_3_	⋯	⋯	0
HSO	⋯	⋯	1
H_2_SO_4_	⋯	⋯	1
SO_2_	3 × 10^9^	⋯	1
SO	⋯	⋯	0
CO_2_	⋯	10^−6^−∼5 × 10^−1^	⋯
SO_4_AER	⋯	⋯	0.01
S_8_AER	⋯	⋯	0.01
HCAER	⋯	⋯	0.01
HCAER2	⋯	⋯	0.01

## Appendix B Alternative Visualization of Model Differences

Figures 16–21 more quantitatively show the differences in atmospheric trace gas abundances for the F-, G-, and K-type host stars, as well as the M5.5eV host star.
